# si-Tgfbr1-loading liposomes inhibit shoulder capsule fibrosis via mimicking the protective function of exosomes from patients with adhesive capsulitis

**DOI:** 10.1186/s40824-022-00286-2

**Published:** 2022-08-19

**Authors:** Yaying Sun, Zhiwen Luo, Yisheng Chen, Jinrong Lin, Yuhan Zhang, Beijie Qi, Jiwu Chen

**Affiliations:** 1 Department of Sports Medicine, Huashan Hospital, Fudan University, Shanghai, China; 2 Department of Sports Medicine, Shanghai General Hospital, Shanghai Jiaotong University, Shanghai, China

**Keywords:** Adhesive capsulitis, Rotator cuff tear, Circulating exosomes, miRNAs, Liposomes

## Abstract

**Background:**

Adhesive capsulitis is a common shoulder disorder inducing joint capsule fibrosis and pain. When combined with rotator cuff tear (RCT), treatments can be more complex. Currently, targeted therapy is lacking. Since adhesive capsulitis is reported to be related to circulating materials, we analyzed the contents and biology of circulating exosomes from RCT patients with and without adhesive capsulitis, in an attempt to developing a targeting treatment.

**Methods:**

Samples from a consecutive cohort of patients with RCT for surgery were collected. Circulating exosomal miRNAs sequencing were used to detect differentially expressed miRNAs in patients with and without adhesive capsulitis. For experiments in vitro, Brdu staining, CCK-8 assay, wound healing test, collagen contraction test, real-time quantitative polymerase chain reaction, and western blot were conducted. Histological and immunofluorescent staining, and biomechanical analysis were applied in a mouse model of shoulder stiffness. The characteristics of liposomes loaded with siRNA were measured via dynamic light scattering or electron microscopy.

**Results:**

Circulating exosomal miRNAs sequencing showed that, compared to exosomes from patients without adhesive capsulitis, miR-142 was significantly up-regulated in exosomes from adhesive capsulitis (Exo-S). Both Exo-S and miR-142 could inhibit fibrogenesis, and the anti-fibrotic effect of Exo-S relied on miR-142. The target of miR-142 was proven to be transforming growth factor β receptor 1 (Tgfbr1). Then, liposomes were developed and loaded with si-Tgfbr1. The si-Tgfbr1-loading liposomes exhibited promising therapeutic effect against shoulder stiffness in mouse model with no evidence toxicity.

**Conclusion:**

This study showed that, in RCT patients with adhesive capsulitis, circulating exosomes are protective and have anti-fibrotic potential. This effect is related to the contained miR-142, which targets Tgfbr1. By mimicking this biological function, liposomes loaded with si-Tgfbr1 can mitigate shoulder stiffness pre-clinically.

**Supplementary Information:**

The online version contains supplementary material available at 10.1186/s40824-022-00286-2.

## Background

Adhesive capsulitis, or idiopathic shoulder stiffness, is a common disorder affecting 2–5% of general population and reaching 40% in diabetic patients [1]. Local inflammation and subsequent fibrosis are classical pathological changes [2]. As a consequence, patients suffer from much shoulder pain and limited joint motion. Many interventions, including physiotherapy plus exercise, anti-inflammatory agents, or even surgical release, can be administered to patients [3], reflecting a complexity of this condition.

Rotator cuff tear (RCT) is another common glenohumeral complaint inducing joint pain and dysfunction especially in elderly [4]. In patients with RCT, the portion of concomitant adhesive capsulitis can be 10% to 40% [5, 6], while in patients with adhesive capsulitis, ~ 30% have concomitant full-thickness RCT [7]. Surgically repairing of the torn rotator cuff has been accepted worldwide for decades, yielding overall satisfactory results [8], but how to treat RCT in patients with adhesive capsulitis is still controversial.

Some suggest to simultaneously treat RCT and adhesive capsulitis during surgery, but rotator cuff repair is a joint-tightening operation, which may exacerbate joint stiffness [9]. Some insist on treating adhesive capsulitis conservatively before conducting surgery for RCT, whereas the prolonged period of physiotherapy may enlarge the size of RCT [10]. Therefore, targeted therapy for adhesive capsulitis is needed.

To maintain homeostasis, our body have evolved self-protective and self-repair mechanisms. For example, after spinal cord injury, OXR1, a neuroprotective protein, is up-regulated [11]; following contusion, multiple lncRNAs are elevated or declined to initiate myogenesis [12]; in case of acute infections, white blood cells elevate and accumulate to the focus against bacteria. Enlightened by this phenomenon, we speculate that, within RCT population, studying the difference between those with and without adhesive capsulitis may help developing therapies.

Current evidence suggests that metabolic and autoimmune factors are related to adhesive capsulitis [13]. Serna et al. announced that materials in the circulation may provide the clue [14]. Among various factors in the blood circulation, exosomes have attracted increasing attention. Secreted by various kinds of cells, exosome is a key member of extracellular vesicles with a size from 30 to 150 nm diameters expressing markers such as Alix, CD63, and CD9 et al. [15].

Enriched in circulation [16], exosome is a valuable biomarker and player in various physical and pathological conditions. Its cargos, especially microRNAs (miRNAs), play a pivotal role [17]. In lung adenocarcinoma, exosomal miR-214 is up-regulated in circulation to promote bone metastasis by activating osteoclasts [18]. In kidney fibrosis, anti-fibrotic miR-483 was elevated in blood and declined in renal tubular epithelial cells, thus creating a pro-fibrotic intra-cellular environment [19].

**Fig. 1:**
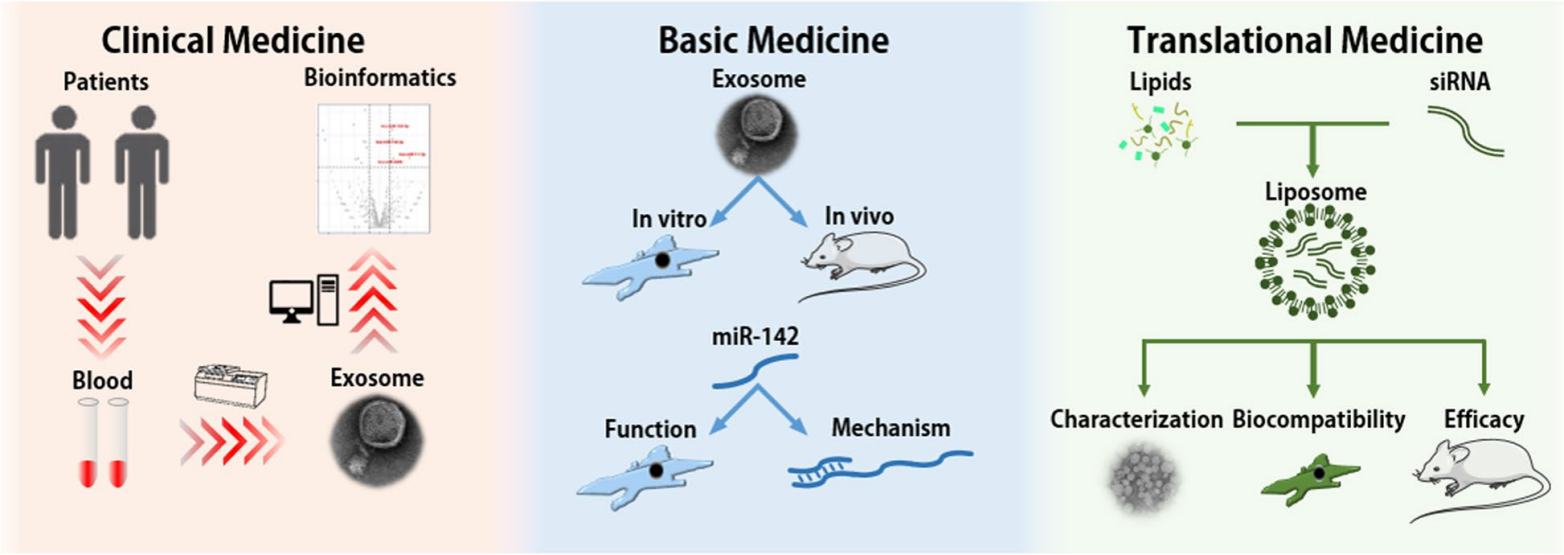
Research design of the current study. First, in the Clinical Medicine section, we collected the samples of patients included and extracted circulating exosomes for miRNAs sequencing. Then, in Basic Medicine section, we organized a series of experiments test the biological function exosomes and the contained miRNA. Finally, in the Translational Medicine Section, enlightened by the biological function of exosomal miR-142, we prepared si-Tgfbr1-loading liposomes and verified the anti-fibrotic effect, providing a potential therapy against adhesive capsulitis

On ground of this, we harvested circulating exosomes from RCT patients with and without adhesive capsulitis, and analyzed the expression profile of exosomal miRNAs. Experiments were then conducted to figure out the biological function of exosomes and the contained miRNAs. Finally, an anti-fibrotic strategy was designed based on the experiments, and the efficacy was tested pre-clinically (Fig. 1).

## Methods and materials

Institutional ethical committee of Huashan Hospital approved the current study (KY-2018–0390). All participants signed informed consent. The research was organized abiding by the Declaration of Helsinki. Animal procedures were approved by the Institutional Animal Care and Use Committee of Shanghai Medical College, Fudan University. The experiments complied the Guide for the Care and Use of Laboratory Animals.

### Patients inclusion and data collection

From March 2019 to October 2019, a consecutive cohort of patients with RCT of full thickness were followed. Patients with pain visual analog scale (VAS) > 7 [20], which reflects much inflammation and are not eligible for operation, were excluded in outpatient. Partial RCT, confirmed during surgery, were also excluded. Demographic data and disease-related data including duration, and range of motion (ROM) of the index shoulder in external rotation at side, flexion, abduction, and internal rotation at the back, were collected. The definition of adhesive capsulitis was passive ROM deficiency greater than 25% or 30° compared to normal/contralateral value in at least two of the three directions (flexion, external rotation at side, and internal rotation at back) [21]. Internal rotation was scored according to Sun et al. [20]. Patients with joint stiffness were named as group S, while those without were named as group NS.

### Sample collection

Fasting blood samples of 15 ml were harvested one day prior to surgery in EDTA-coated tubes. Collected after centrifugation at 1000 × g for 10 min, plasma was then transferred into another tube, and further centrifuged 15 min at 2500 × g at 4 °C. Samples were store at − 80 °C before use.

For exosomes separation, plasma was centrifuged (Optima XPN-100 ultracentrifuge) at 10,000 g for 0.5 h first, filtered using 0.22 μm filter (Merck Millipore), and then ultracentrifuged in another tube at 100,000 g for 70 min to pellet vesicles at 4 °C. Washing once using 1 × PBS, exosomes were and re-suspended in PBS and preserved at − 80 °C.

Exosomes from group S was entitled as Exo-S, while the counterpart was named as Exo-NS. For miRNA sequencing, three samples from each group was individually analyzed without mixture. For experiments in vitro and in vivo, exosomes were mixed together prior to intervention. During surgery, the joint capsule samples of patients were also collected for other measurements or to harvest capsular derived fibroblasts (CDFs) based on published protocol [22].

### Electron microscopy

Samples were assessed by scanning electron microscope (SEM, TESCAN) or transmission electron microscope (TEM, Tecnai). General procedures of SEM were in line with previous protocols [20]. Briefly, 0.1 ml exosomes suspension was frozen for 12 h at -20 °C, and subjected to vacuum drying. Sample was put onto the freezing glue on the sample platform. Finally, exosomes sample was coated with gold and observed by SEM. For TEM, exosomes were set on copper grid coated with 0.125% Formvar in chloroform. Grid was stained with 1% uranyl acetate in dd-water and samples were imaged using the TEM. Liposomes were measured in the same manner. TEM of isolated exosomes was conducted as aforementioned [23].

### Nanoparticle tracking analysis (NTA)

Size and concentration of exosomes were quantified using the Flow Nanoanalyzer (High Sensitivity Flow Cytometry, China) with data processing [24]. 10 μl of one sample was diluted with PBS and processed by Nanoanalyzer. NTA software recorded the whole data with settings as follows: Laser setting: 5/40 mW 488; Min Width: 0.3 ms; Threshold: 75.6 9.8 1.7; SS Decay: 10 percent; Sample pressure: 1.0 Kpa.

### Flow cytometry analysis (FACS)

General procedures of FACS were according to the MIFlowCyt EV Reporting Framework [25]. Exosomes markers were probed by FACS using Exo-Flow capture kit followed with the manufacturer’s protocol [26]. Data analysis was performed on FlowJo software (Version 10). Antibodies used were shown in Table S1 (Additional File 1).

### Exosomal microRNAs sequencing

Sequencing services were provided by Personal Biotechnology Co., Ltd. Shanghai, China. RNA from Exo-NS and Exo-S (*n* = 3, respectively) was extracted by miRNeasy Kit (Qiagen, Hilden, Germany). Quality and purity were evaluated using Qubit (Life Technologies, USA) and Agilent 2200 TapeStation (Agilent Technologies). Sequencing analysis of exosomal small RNA was conducted on Illumina NextSeq 500 platform. |log_2_ (fold change)|≥ 1 with *P* < 0.05 was entitled as differential expression.

### Bioinformatic analysis and target prediction

Gene Ontology (GO), Kyoto Encyclopedia of Genes and Genomes (KEGG) pathway enrichment analysis and annotation were performed by DAVID [27]. Targetminer, miRWalk, and mirDIP were used for the target gene of miRNA.

### Cell culture

Two kinds of fibroblasts, CDFs and NIH3T3 cells were used. Both cells were maintained in high-glucose DMEM medium (Thermo Fisher Scientific) plus 10% Certified Fetal Bovine Serum, FBS (VivaCell, Shanghai, China, C04001-500) + 1% penicillin/streptomycin. Harvested CDFs were considered as passage 0. Cells were passaged when confluence reaching ~ 90%. CDFs at passage 3 were utilized. Cells were cultured in humidified incubator with 5% CO_2_ atmosphere at 37℃.

### In vitro PKH67 tracing for exosomes

Exosomes were labeled with PKH67 using Green Fluorescent Labeling Kit (Sigma, Aldrich) based on the protocol. Exosomes or PBS were stained by PKH67 dye for 5 min at rt. The labeling process was halted with 1 ml 1% BSA. Next, re-purified exosomes were ultracentrifugated in PBS for 0.5 h, and co-cultured with fibroblasts for 12 h. After incubation, the culture plate was rinsed with PBS in triplicate. Fibroblasts were fixed in 4% paraformaldehyde and stained by Dapi (Beyotime, China). The uptake of labeled exosomes by cells was examined by fluorescence microscope (ECHO Revolve, America).

### Cell interventions

When reaching 80% confluence, growth medium were replaced serum-free medium. Transforming growth factor-β of 20 ng/ml (Sigma, St Louis, USA) was stimulated for 24 h. The working concentration was 20 or 50 μg/ml for Exo-NS and Exo-S.

The transfection was conducted according to protocol. Small interfering RNAs (siRNA) were acquired from RiboBio (Guangzhou, China) with transfection agents. One si-Tgfbr1 (Homo sapiens) and three si-Tgfbr1 (Mus musculus) were obtained. To overexpress or knockdown miR-142-3p (miR-142), miR-142 mimics (10 nM) or inhibitors (50 nM) and NC mimics were also purchased from RiboBio (Sequences in Table S2, see Additional file 1).

### Brdu staining and CCK-8 assay

Cell proliferation was detected by 5-Bromo-2′-deoxyuridine (Brdu) incorporation assay kit (Cell Signaling Technology, USA). 24 h following interventions, cells treated with Brdu agent for another 12 h. Then cells were fixed, washed, and incubated with anti-Brdu antibody for 60 min. Dapi was applied to stain nucleus. Cell proliferation was calculated using the number of Brdu + cells/number of Dapi. Cell viability was assessed by Cell counting kit-8 (CCK-8, Beyotime, China) [28].

### Wound healing and Collagen contraction test

Wound healing was conducted to observe cell migration via scratching a straight line in cells with 80% confluence in 6-well plate. 24 h after interventions, the scratch was observed microscopically. Migration was calculated by the percentage of wound-healing rate (migrated distance/original distance), and normalized to the distance of group negative control (NC). Collagen contraction analysis was conducted as mentioned elsewhere [29], and quantified with ImageJ software (NIH, USA).

### Western blot

Proteins of interest were examined as previously mentioned [30]. The information of antibodies were recorded in Supplementary Table 1. Specifically, albumin, which only present in plasma but not in exosomes, serves as NC gainst exosomes. The expression of proteins were quantified by ImageJ software and normalized by group NC (*n* = 4/group).

### Real-time quantitative polymerase chain reaction (qPCR)

Exosomal miRNAs were extracted by a commercial Kit (Invitrogen). Then a Mir-X™ kit (Takara, Japan) and SYBR® Premix Ex Taq™ II (Takara) were used for measuring the levels of miR-7–1, miR-4488, miR-142, and miR-122 in exosomes. Cel-miR-39 (Ambion) served as reference.

To validate the expression of intra-cellular RNAs, total RNA was extracted using the Trizol reagent (Invitrogen) and quantified by Nanodrop (Thermo Scientific, Waltham, USA). The level of miRNAs was quantified by Stem loop qPCR (TaqMan) with U6 as reference [31]. TB GreenTM Premix Ex TaqTM II (Takara; RR820A) was used for determine the level of mRNAs with GAPDH as reference. miRNA qPCR primers (RiboBio, China) and mRNA qPCR primers (Sangon, China) were listed in Table S3 (see Additional File 1). Comparative Ct method (2^−ΔΔCt^) was used to obtain expression (*n* = 3). All values were normalized by group NC and then compared.

### Dual-Luciferase reporter assay

CDFs were transfected with luciferase vectors of wild-type or mutant 3ʹ-Untranslated Region (3’-UTR) of Tgfbr1, as well as miR mimics by Lipofectamine 3000 (Invitrogen). Luciferase activity was quantified by Dual-Luciferase Reporter Assay System (Beyotime) after transfection for 48 h.

### si-Tgfbr1-loading liposome preparation and characterization

Dissolved in ethanol, the components of liposomes, i.e., C12-200, cholesterol, DSPC, and mPEG-DMG reached a molar ratio of 50:38.5:10:1.5, and then mixed with siRNA dissolved in citrate buffer (10 mM, pH = 3) via vortexing. Free siRNA was discarded via ultrafiltration centrifugation. Encapsulation efficiency was calculated using Quant-iT™ RiboGreen® RNA assay kit (Molecular Probes, UK) via spectrofluorometer. Excitation wavelength was 480 nm and emission wavelength was 520 nm [32]. Acquired liposomes diluted in PBS prior to utilization. Entrapment efficiency was assessed by RiboGreen assay. Hydrodynamic diameter, zeta potential, polydispersity index, and stability were quantified by dynamic light scattering (Malvern Zetasizer, Nano-ZS, UK) [33]. Morphology was observed by TEM. Biocompatibility of liposomes was tested using CCK-8 in NIH3T3 cells co-cultured for 24 h.

### Establishment of shoulder stiffness model, intervention, and biomechanical analysis

Male C57/6 J mice of ~ 10 weeks were used in the current study. Shoulder stiffness model was created via the immobilization of left shoulder [34, 35]. One week after procedure, weekly intra-articular injection of 50 μl exosomes (20 μg/ml and 50 μg/ml) or liposomes (200 nM) was conducted using micro-syringe (flowchart in Fig. S1, see Additional file 1).

All animals could move freely in cage after operation. Mice with sham surgery was named as group Control. Mice with immobilization surgery plus PBS injection was named as group Model. Mice with immobilization surgery plus agent injection were named according to the agent used. Preliminary data indicated that five animals were needed in each group to reach statistical significance between group Model and group Model plus treatments for an improvement of 20° in ROM (power of 90% with type I error level of 0.05), therefore six animals were allocated into one group. Animals with immobilization were randomly allocated to different groups via random numbers.

Three weeks after model establishment, mice were euthanized by CO_2_ overdose and shoulders were harvested. ROM was calculated with the method of Oki. [36]. All measurements were conducted by a researcher blind to group allocations.

In addition, to assess liposome toxicity in vivo, another 12 mice with immobilization operations were included. Among them, three received no injection and were sacrificed 3 weeks after surgery. Nine mice received weekly intra-articular injection of si-Tgfbr1-loading liposomes at one week after surgery and were sacrificed at 3, 7, and 14 days after the first injection. Major organs were collected to evaluate any morphological abnormalities.

### Liposome uptake/tracing

To verify the uptake of liposomes in vitro and in vivo, empty liposomes were labeled with 3,3’-dioctadecyloxacarbocyanine perchlorate (DiO) (ab189809, Abcam, USA) or DiR (ab189810, Abcam, USA) at 37 °C for 20 min according to the protocol. Liposomes were then co-cultured with NIH3T3 cells for 30 min or 1 h, or injected into shoulders of mice model for 24 h (*n* = 3) or 48 h (*n* = 3). These mice were sacrificed to harvest shoulder samples into OCT (stored at -80 °C), or directly subject to in vivo imaging with the IVIS software (Living Image Software) for the evaluation of fluorescence intensity.

### Histological and immunofluorescent analysis

Samples were decalcified, dehydrated, and embedded in paraffin wax. The method of HE was according to the published study [37]. For masson staining, the procedures were conducted according to the commercially available protocol of Masson staining kit (Solarbio, China). Number of nucleus infiltrated in joint capsule was obtained in area of 25 μm*25 μm with 6 random measures to get a mean number by ImageJ 7.0 (NIH, Bethesda, MD, USA). The thickness, also obtained by 6 measures in each sample, was normalized to the value of group NS. Immunofluorescent staining in vivo and in vitro were published elsewhere [29, 38]. Images were captured by microscopically (Olympus, X71).

In terms of shoulder samples used for liposome tracing, slices were blocked in 5% BSA with 0.5% Triton-X-100 (Solarbio, Beijing, China) for 60 min. Slices were then stained Dapi and observed immunofluorescently.

### Statistical analysis

Comparisons were conducted by SPSS 18.0 software. Continuous data was shown as mean ± SD. Student t-test or ANOVA and post hoc test was used to determine intergroup difference. Difference in categorical data was assessed by Fisher exact test or Chi-square test. 2-tail *P* < 0.05 was entitled as statistical significance.

## Results

### Characteristics of exosomes derived from RCT patients with and without adhesive capsulitis

Based on inclusion and exclusion criteria, 51 patients were included, among which 9 were in group S. Detailed demographic data and passive ROMs was shown in Table S4 (see Additional File 1). There was no significant difference between group NS and group S regarding gender, age, predominant side, height, and weight. On the other hand, passive ROMs of group S were significantly lower than group NS, reflecting a stiffened status of joint. Immunofluorescently, significantly more Col 1 and α-SMA expressed in capsule of group S than of group NS (Fig. S2, Additional File 1).

**Fig. 2:**
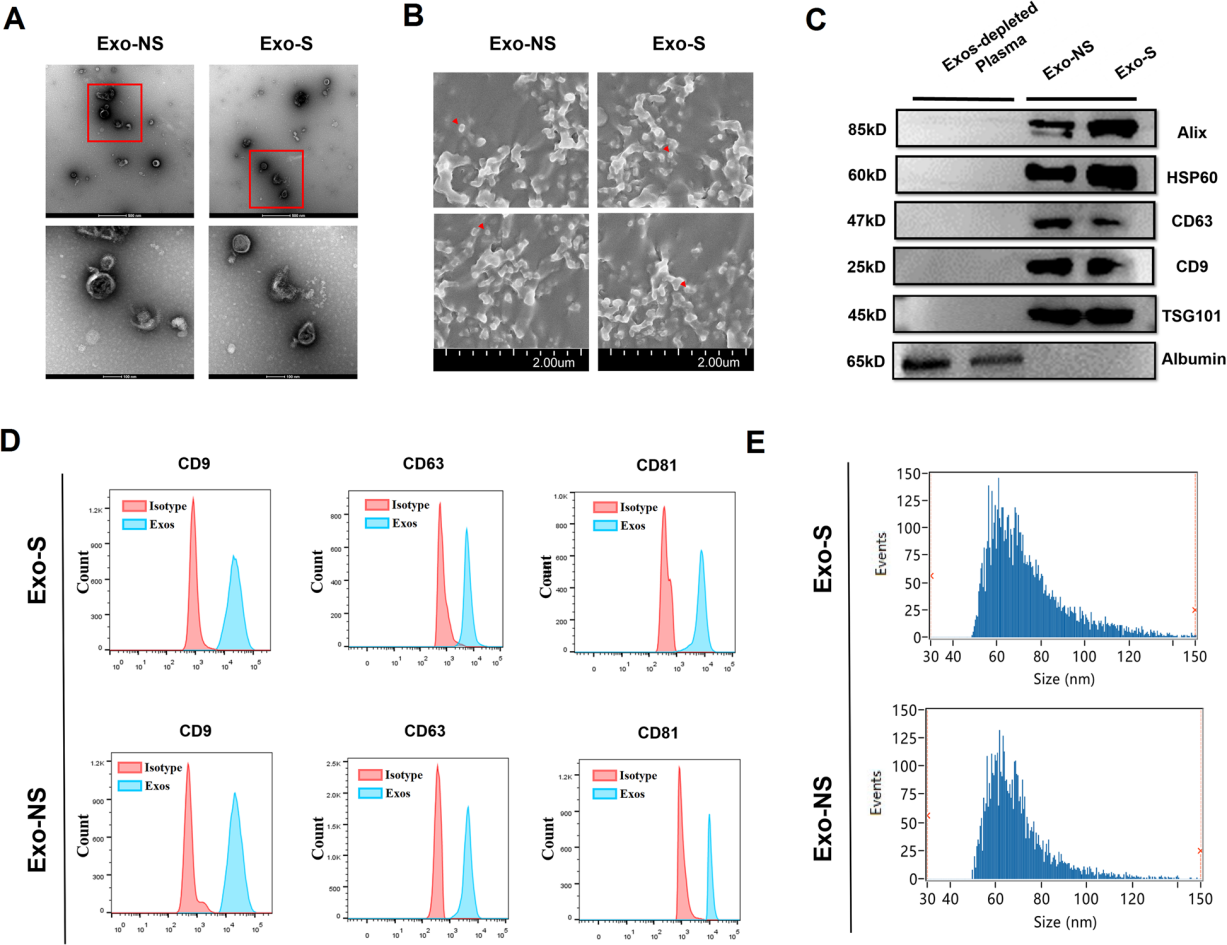
Characterization of Exo-NS and Exo-S. **A** and **B** Representative TEM (bar = 500 and 100 nm) and SEM (bar = 2 μm) image of exo-NS and Exo-S; **C** Western blots of positive and negative markers of Exo-NS and Exo-S; **D** Flow cytometry of exosomes markers; **E** Particle size distribution of circulating exosomes quantified by NTA

Exo-NS and Exo-S were then isolated. Exosomes from both groups exhibited a spherical structure observed under TEM (Fig. 2A) and SEM (Fig. 2B). Western blot detected the expression of Alix, HSP60, CD63, CD9, and TSG101 in exosomes rather than in Exo-depleted plasma, and albumin was only pinpointed in plasma (Fig. 2C). Flow cytometry also identified positive expression CD9, CD63, and CD81 of exosome samples (Fig. 2D). NTA showed that the median diameter of Exo-NS and Exo-S was 73.92 nm and 69.25 nm, respectively (Fig. 2E). These data validated a qualified collection of exosomes.

### Exo-S inhibited fibrogenesis in vitro

**Fig. 3:**
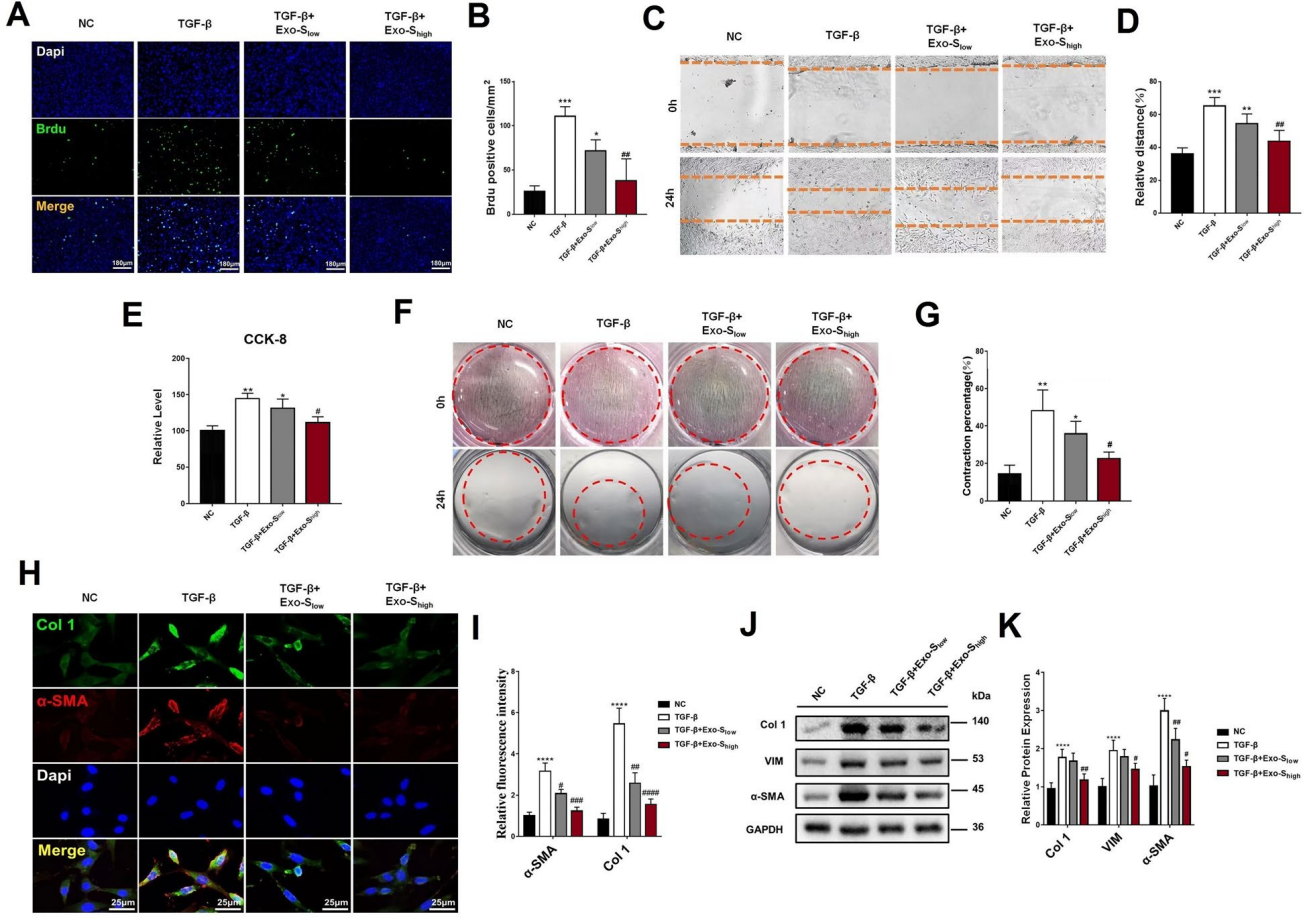
Exo-S relieved fibrogenesis of CDFs. **A** and **B** Proliferation was detected by Brdu assay at 24 h following TGF-β culturing or concomitant Exo-S intervention with different concentrations, and quantification. Bar = 180 μm; **C** Migration ability of CDFs and quantification; **E** Viability of CDFs determined using CCK-8 assay. **F** and **G** Collagen contraction of CDFs (red dotted circle illustrated the collagen) and quantification; **H** and **I** Immunofluorescent staining of Col 1 and α-SMA in CDFs (bar = 25 μm) and quantification; **J** and **k** Protein level of fibrotic markers in CDFs and quantification. *: *P* < 0.05 compared to group NC; **: *P* < 0.01 compared to group NC; ***: *P* < 0.001 compared to group NC; #: *P* < 0.05 compared to group TGF-β; ##: *P* < 0.01 compared to group TGF-β; ###: *P* < 0.001 compared to group TGF-β; ####: *P* < 0.0001 compared to group TGF-β

After confirming that both Exo-NS and Exo-S were successfully internalized by CDFs (Fig. S3, see Additional File 1), the biological function of both exosomes was detected. Similar to previous reports [39], TGF-β, the robust pro-fibrotic factor, accumulated in joint capsule of stiffened shoulder (Fig. S4, see Additional File 1). Therefore, we used TGF-β as the stimuli in vitro. Exo-S significantly disturbed CDFs proliferation (Fig. 3A and B), migration (Fig. 3C and D), and viability (Fig. 3E) under TGF-β stimulation. Collagen contraction assay suggested that Exo-S inhibited the contractile ability of CDFs induced by TGF-β (Fig. 3F and G). Immunofluorescent staining revealed that the up-regulation of Col 1 and α-SMA in CDFs by TGF-β was suppressed by Exo-S (Fig. 3H and I), which was also verified by western blot (Fig. 3J and K). On the other hand, Exo-NS did not disrupt TGF-β-induced activation of CDFs (Fig. S5, see Additional File 1).

Consistently, Exo-S was successfully internalized by NIH3T3 fibroblasts (Fig. S6, see Additional File 1) and inhibited the fibrogenesis (Fig. S7, see Additional File 1). These evidence implied an anti-fibrotic effect of Exo-S.

### Exo-S inhibited capsular fibrosis in vivo

**Fig. 4:**
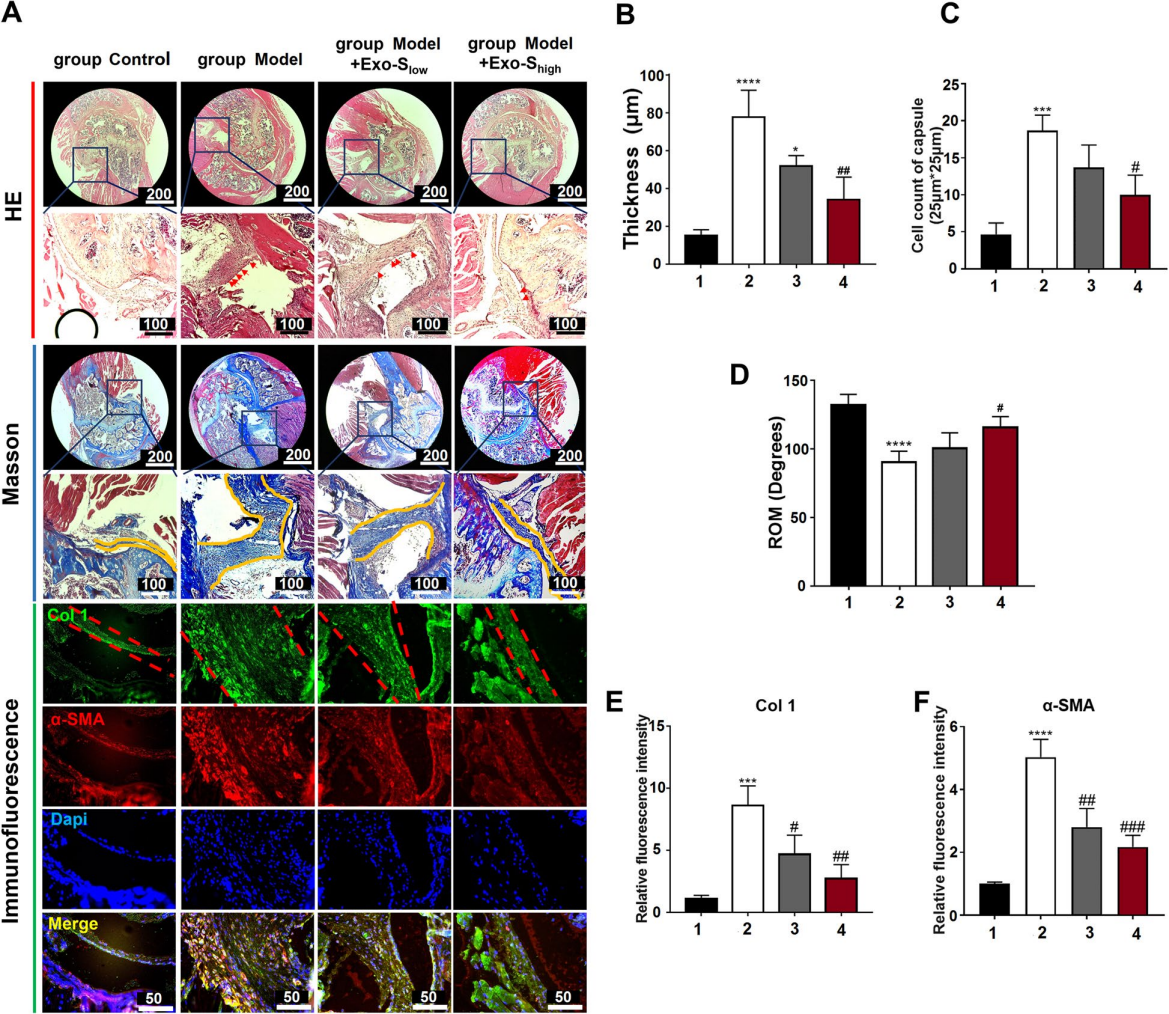
Exo-S inhibited shoulder stiffness in vivo. **A** Typical picture of joint capsule on HE and Masson staining (bar = 200 or 100 μm), as well as immunofluorescent staining for Col 1 and α-SMA (bar = 50 μm). Red arrow indicated nucleus, yellow line indicated the borders of joint capsule; **B** The quantification of capsule thickness among different groups; **C** Cell counts per 25 μm*25 μm; **D** passive ROM of the index shoulders; **E** and **F** Quantification of α-SMA and Col 1 in capsule samples. 1: group Control; 2: group Model; 3: group Model + Exo^low^; 4: group Model + Exo^high^. *: *P* < 0.05 compared to group Control; ***: *P* < 0.001 compared to group Control; ****: *P* < 0.0001 compared to group Control; #: *P* < 0.05 compared to group Model; ##: *P* < 0.01 compared to group Model; ###: *P* < 0.001 compared to group Model

Representative HE staining, Masson staining, Col 1, and α-SMA staining of mice shoulders were shown in Fig. 4A. Immobilized joints had prominent cell infiltration in capsule (Fig. 4B). Masson staining noted thickened capsule in group Model and Exo-S injection groups than in group Control, but the thickness declined with Exo-S injection (Fig. 4C). Accordingly, passive ROM was restored by Exo-S (Fig. 4D). Similarly, Exo-S reduced Col 1and α-SMA accumulation in joint capsule (Fig. 4E and F, respectively). These data implied that Exo-S exhibited anti-fibrotic potential in vivo.

### miR-142 was significantly up-regulated in circulating exosomes from RCT patients with adhesive capsulitis

**Fig. 5:**
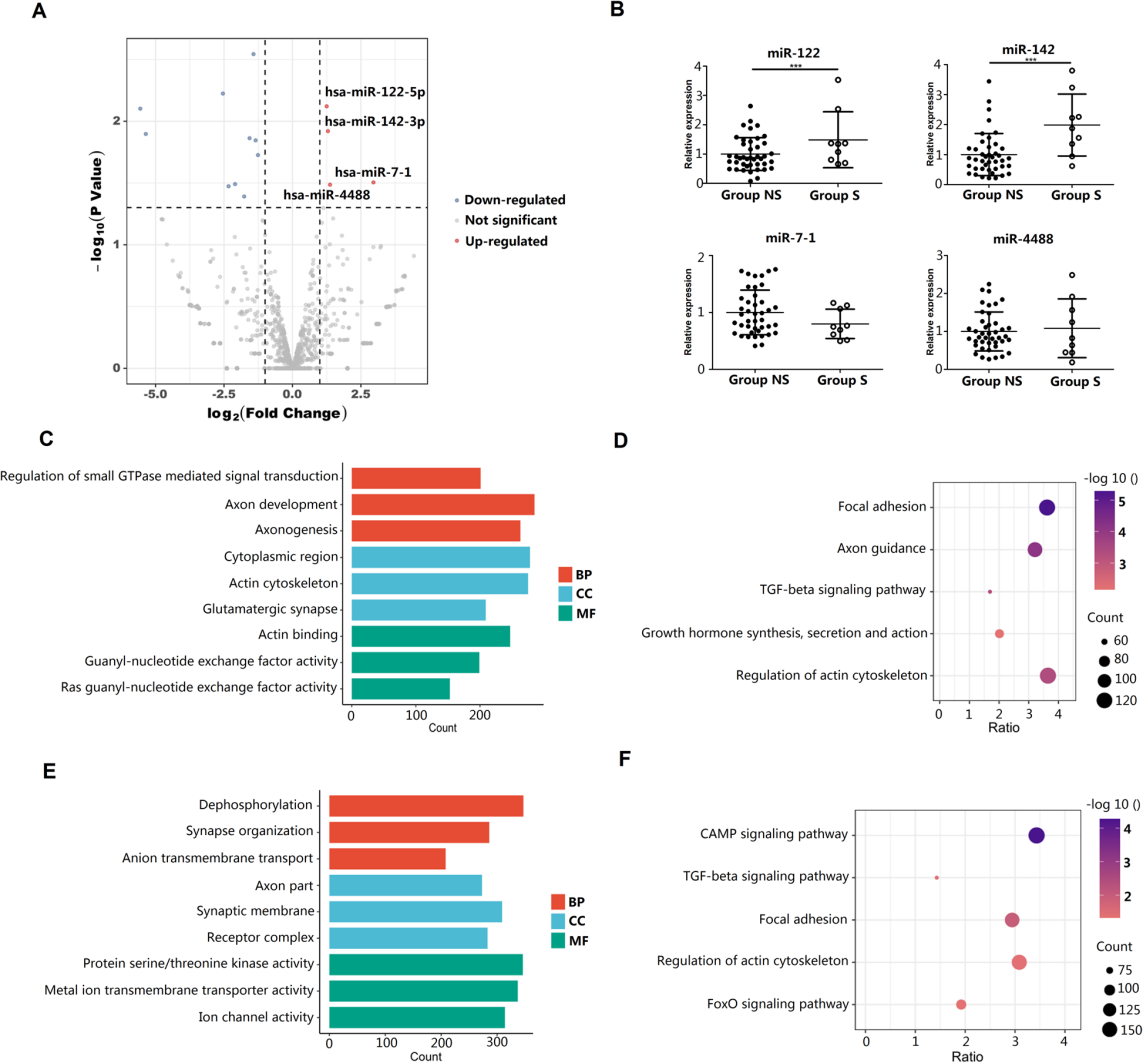
miRNAs expression profile in Exo-S compared to Exo-NS. **A** Volcano plot showed miRNAs of differential expression between Exo-S and Exo-NS. Red: up-regulated expression; **B** qPCR verification of miRNAs of interest; **C** GO enrichment for miR-142; **D** Bubble plot of KEGG enrichment for miR-142; **E** GO enrichment analysis for miR-122; **F** Bubble plot of KEGG enrichment for miR-122. ***: *P* < 0.001 compared to group NS

Next, exosomal miRNAs sequencing was conducted between Exo-S and Exo-NS (GSE182896). Four miRNAs were significantly up-regulated in Exo-S compared to Exo-NS, i.e., miR-7–1-3p, miR-4488, miR-142-3p, and miR-122-5p (Fig. 5A). qPCR analysis verified that, compared to that of Exo-NS, miR-142 and miR-122 were significantly up-regulated in Exo-S (Fig. 5B).

GO analysis suggested that miR-142 (Fig. 5C) and miR-122 (Fig. 5E) participated in various molecular functions, cellular components, and biological processes, and KEGG pinpointed that TGF-β signaling was among the various pathways targeted by miR-142 (Fig. 5D) and miR-122 (Fig. 5F). This information suggested that miR-142 and miR-122 might be engaged in the anti-fibrotic process. Considering that miR-142 had an elevation of approximately 100% while miR-122 only had ~ 50% (Fig. 5B), the former was chosen for further investigations.

### miR-142 inhibited fibrogenesis by targeting Tgfbr1

Then we questioned whether miR-142 could inhibit fibrogenesis. miR-142 mimics inhibited the effect of TGF-β on cell proliferation (Fig. S8A and S8C, see Additional File 1), migration (Fig. S8B and S8D, see Additional File 1), and viability (Fig. S8E, see Additional File 1), while miR-142 inhibitor had opposing effect. Western blot found that p-Smad2/3 was significantly up-regulated by TGF-β and was inhibited by miR-142 mimics (Fig. S8F and S8G, see Additional File 1). Collagen contraction test further proved that, miR-142 abolished TGF-β mediated contraction ability of CDFs, which was enhanced by miR-142 inhibitor (Fig. S8H and S8I, see Additional File 1).

**Fig. 6:**
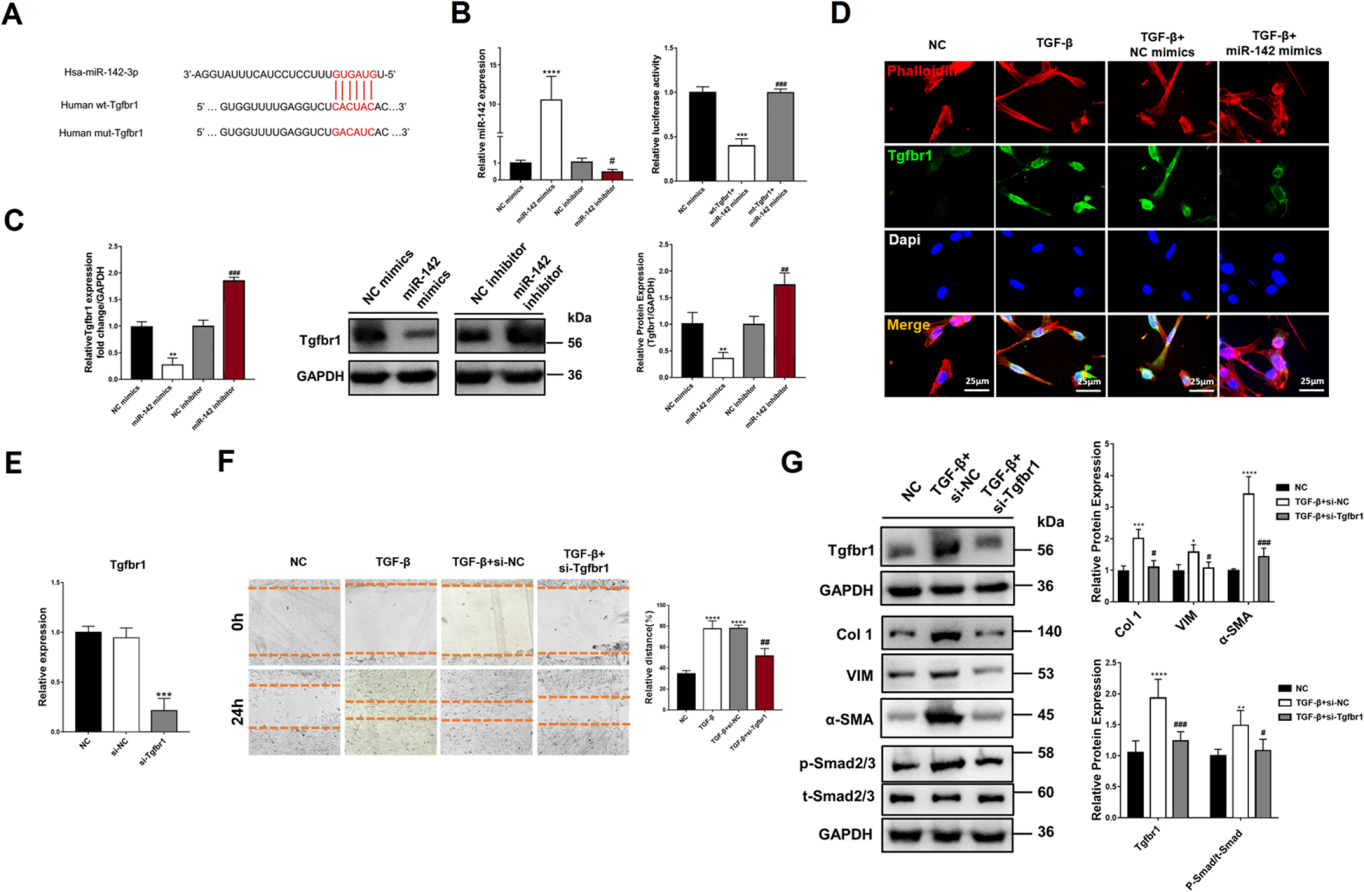
Tgfbr1 was directly targeted by miR-142. **A** The binding sequence between Tgfbr1 3’-UTR and miR-142. Mutation was generated at the complementary site of 3’-UTR and miR-142 seed region; **B** miR-142 mimics or inhibitor significantly changed the intra-cellular level of miR-142, and the luciferase activity of wild-type Tgfbr1 3’-UTR but not the mutant-type was significantly inhibited by miR-142 mimics. **C** Gene and protein level of Tgfbr1 in CDFs with different interventions; **D** Immunofluorescent staining of Tgfbr1 in CDFs under different stimulations. Bar = 25 μm; **E** The efficacy of gene knock-down of Tgfbr1 in CDFs determined by qPCR. **F** Migration CDFs in different groups and quantification; **G** The level of proteins of interest in CDFs with TGF-β intervention and Tgfbr1 knock-down determined by western blot and quantification. **: *P* < 0.01 compared to group NC mimics or group NC; ***: *P* < 0.001 compared to group NC mimics or group NC; ****: *P* < 0.0001 compared to group NC mimics or group NC; #: *P* < 0.05 compared to group NC inhibitor or group TGF-β + si-NC; ##: *P* < 0.01 compared to group NC inhibitor or group TGF-β + si-NC; ###: *P* < 0.001 compared to group NC inhibitor or group TGF-β + si-NC

The target genes were predicted online. Tgfbr1, the membrane receptors of TGF-β, were identified as a potential target of miR-142. The predicted binding site of miR-142 on Tgfbr1 were shown in Fig. 6A. Luciferase reporter assay showed that miR-142 overexpression disturbed the luciferase activity of Tgfbr1 wild-type rather than mutant (Fig. 6B). qPCR and western blot indicated that miR-142 mimics significantly up-regulated and inhibitor down-regulated the level of miR-142 in CDFs (Fig. 6C). Correspondingly, the fluorescent intensity of Tgfbr1 was inhibited by miR-142 mimics and up-regulated by miR-142 inhibitor (Fig. 6D).

To validate the function of Tgfbr1, this gene was knocked down in CDFs (Fig. 6E). Consistently, siRNA retarded the phosphorylation of Smad2/3, as well as Col 1, VIM, and α-SMA (Fig. 6F). As expected, the migration and viability of CDFs were suppressed by Tgfbr1 inhibition (Fig. 6G).

### The therapeutic effect of Exo-S depended on miR-142

The relationship between Exo-S and miR-142 was investigated. The prohibition of Exo-S on cell proliferation (Fig. S9A and S9B, see Additional File 1) and viability (Fig. S9C, see Additional File 1) was reversed by miR-142 inhibitor. The expression of α-SMA was also restored by miR-142 inhibitor (Fig. S9D and S9E, see Additional File 1).

Finally, we examined the level of miR-142 and Tgfbr1 in shoulder capsule samples. Not surprisingly, the level of miR-142 in group S was significantly lower than that of group NS, while Tgfbr1 had a reversed expression (Fig. S10, see Additional File 1). Therefore, it was a reasonable speculation that, to counteract shoulder capsule fibrosis, circulating exosomes were generated with abundant miR-142 to inhibit aberrant Tgfbr1 expression of the joint capsule.

### Generation of siRNA-loaded liposomes to mimic the therapeutic function of Exo-S

The above clinical and laboratory data unveiled that both miR-142 and Tgfbr1 could be potential targets for treating adhesive capsulitis in patients with RCT. Considering that miRNAs have multiple targets and may induce other effects, Tgfbr1 was selected as our candidate.

First, we obtained three si-Tgfbr1 and verified the most effective one (Fig. S11A and S11B, see Additional File 1). This siRNA could significantly inhibit cell proliferation, fibrotic markers expression, and migration ability up-regulated by TGF-β (Fig. S11C to S11G, see Additional File 1).

**Fig. 7:**
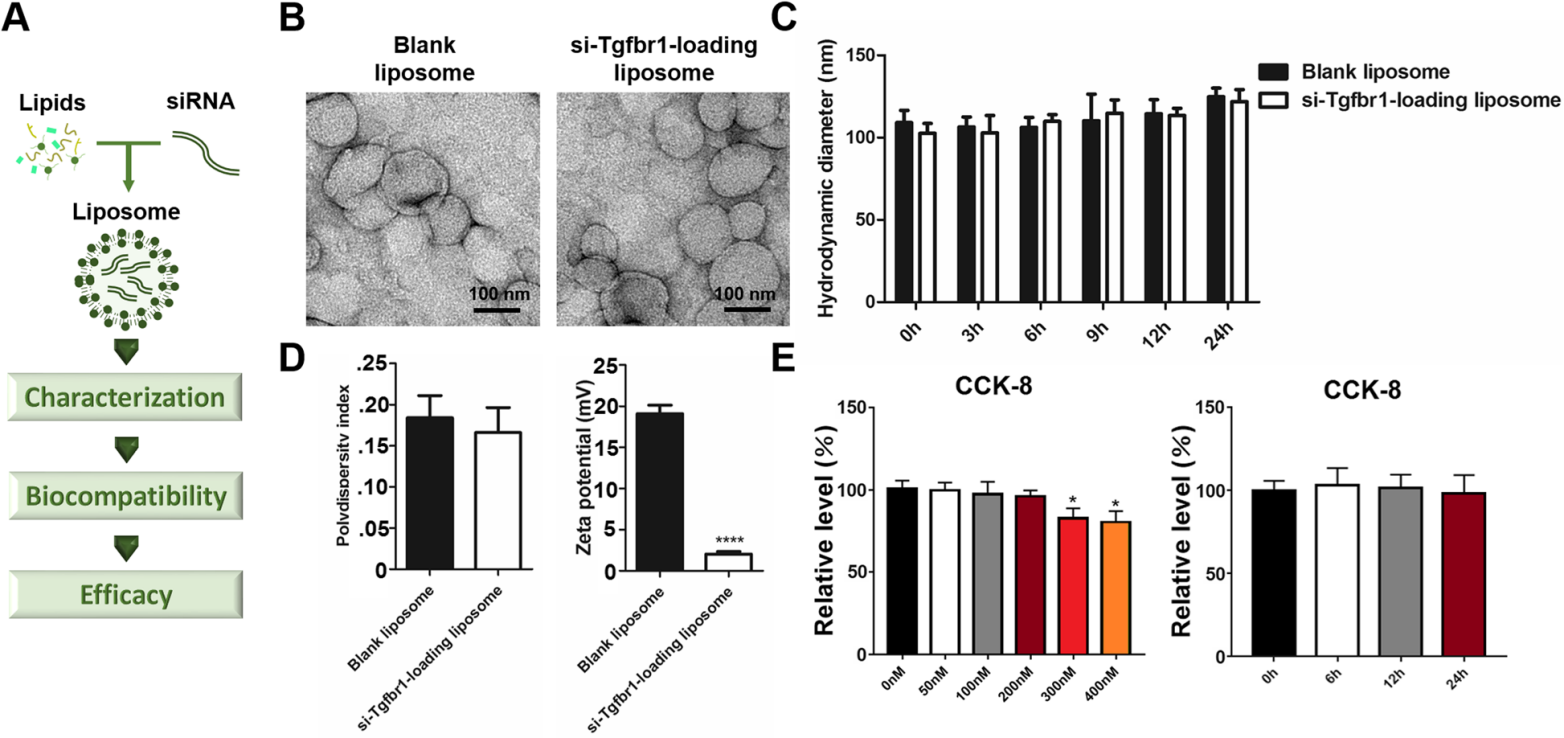
Construction of si-Tgfbr1-loading liposomes and evaluations in vitro. **A** flowchart of liposome preparation and evaluation; **B** TEM imaging of blank liposome and si-Tgfbr1-loading liposomes. Bar = 100 nm; **C** stability of liposomes in PBS for 24 h; **D** Polydispersity index and Zeta potential of liposomes; **E** Toxicity of liposomes evaluated by CCK-8 assay in vitro. NIH3T3 cells were cultured with si-Tgfbr1-loading liposomes under gradient concentrations for 12 h or under 200 nM for 24 h. *: *P* < 0.05; ****: *P* < 0.0001

Liposomes are capable of protecting drugs from degradation/clearance with low toxic effects [40], therefore this agent was prepared to carry and deliver si-Tgfbr1. The characterization, biocompatibility, and pre-clinical efficacy were sequentially tested (Fig. 7A).

First, the DiO-labeled blank liposomes was observed to be successfully uptake in vitro (Fig. S12, see Additional File 1) and in vivo within the first 24 h, and stayed at the location of joint capsule for at least 2 days (Fig. S13, see Additional File 1). Then, liposomes were loaded with si-Tgfbr1 and reached an encapsulation efficiency of 96.52 ± 0.52%. Morphology of the generated liposomes was shown in Fig. 7B. The blank liposomes had a diameter of 108.91 ± 6.96 nm, while that of si-Tgfbr1-loading liposomes was 102.59 ± 5.27 nm. Both liposomes maintained stability for 24 h (Fig. 7C). Polydispersity inidex and Zeta potential of blank liposome was 0.184 ± 0.024 and 19.08 ± 0.93 mV, respectively; and was 0.166 ± 0.027 and 2.02 ± 0.29 mV for si-Tgfbr1-loading liposomes, respectively (Fig. 7D). CCK-8 indicated that when si-Tgfbr1 within liposomes reached a concentration of 200 nM, no significant decline of NIH3T3 viability was observed for at least 24 h (Fig. 7E).

**Fig. 8:**
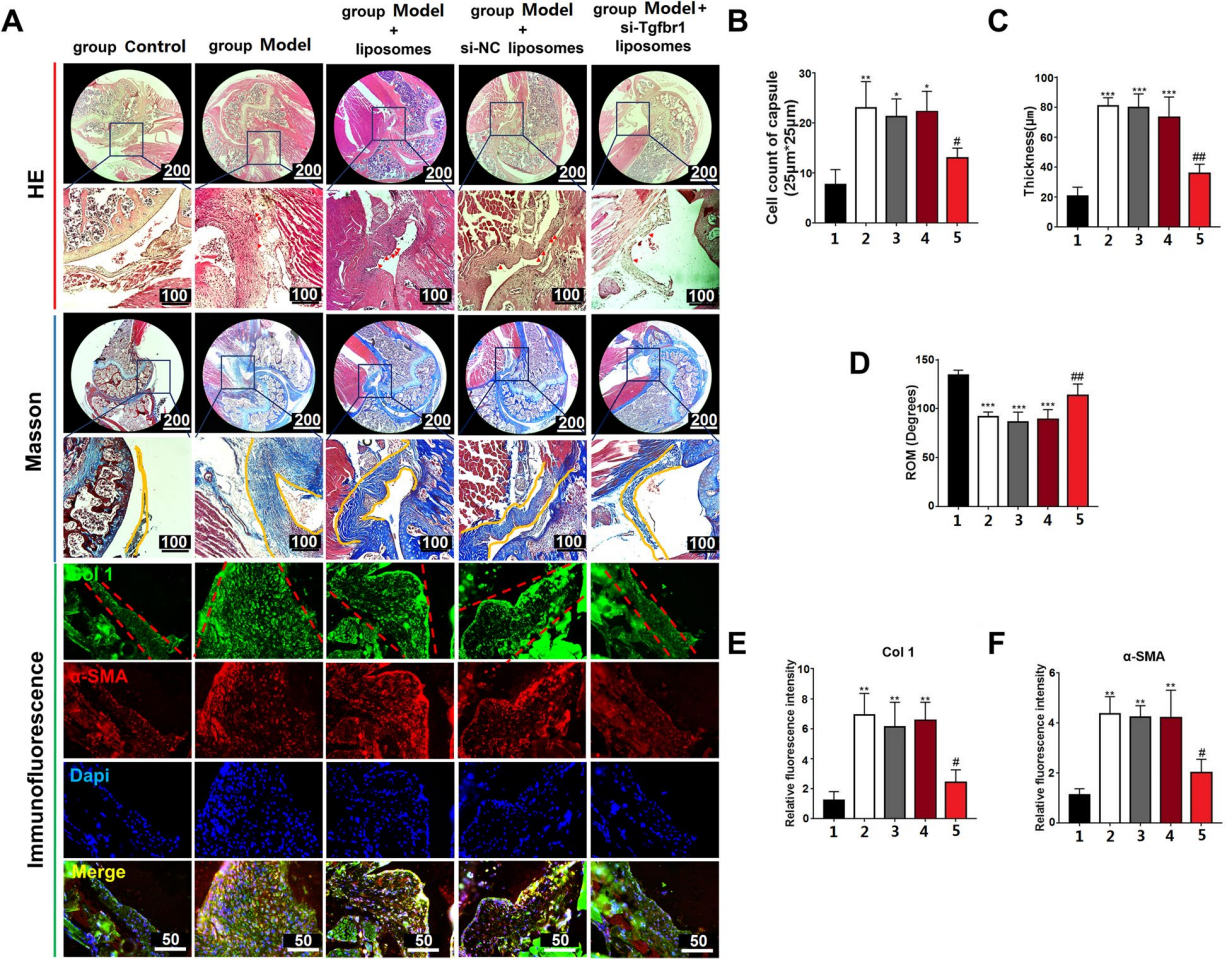
si-Tgfbr1-loading liposomes inhibited shoulder stiffness in vivo. **A** Typical picture of joint capsule on HE and Masson staining (bar = 200 or 100 μm), as well as immunofluorescent staining for Col 1 and α-SMA (bar = 50 μm). Red arrow indicated nucleus, yellow line indicated the borders of joint capsule; **B** The quantification of capsule thickness among different groups; **C** Cell counts per 25 μm*25 μm; **D** passive ROM of the index shoulders; **E** and **F** Quantification of α-SMA and Col 1 in capsule samples. 1: group Control; 2: group Model; 3: group Model + liposomes; 4: group Model + si-NC liposomes; 5: group Model + siTgfbr1 liposomes. *: *P* < 0.05 compared to group Control; **: *P* < 0.01 compared to group Control; ***: *P* < 0.001 compared to group Control; #: *P* < 0.05 compared to group Model; ##: *P* < 0.01 compared to group Model

Finally, safety and therapeutic effect of si-Tgfbr1-loading liposomes with a concentration of 200 nM was tested in vivo. Compared to the organs from mice without liposome injection, major organs from those with injections had no significant morphological abnormalities, indicating limited toxicity (Fig. S14, see Additional File 1). Regarding shoulder stiffness, si-Tgfbr1-loading liposomes significantly inhibited cell infiltration in shoulder capsule, retarded capsule thickening, restored passive ROM, and decreased Col 1 and α-SMA accumulation (Fig. 8). These data together supported the potential of si-Tgfbr1-loading liposomes in treating adhesive capsulitis.

## Discussion

Our data above confirmed that, among patients with RCT, circulating exosomes from those with adhesive capsulitis had anti-fibrotic potential. This effect was related to, at least partially, the contained miR-142. By targeting Tgfbr1, miR-142 blocked TGF-β/Smad signaling pathway to alleviate shoulder stiffness. Then, to inhibit Tgfbr1 expression, we constructed a siRNA-loading liposome and proved that this artificial agent, mimicking the protective function of these circulating exosomes, had therapeutic potential against adhesive capsulitis.

During fibrogenic process, TGF-β signaling binds with Tgfbr2 and activates Tgfbr1, recruiting and phosphorylating Smad2/3 to initiate fibrogenesis [41]. This pathway has a positive feed-back loop that TGF-β can up-regulate the level of Tgfbr1/2 [42]. On ground of this, therapies are developed to disrupt this circle. For example, by inhibiting EZH2, the interactor of Smad3, Li et al. abolished fibroblast activation in hypertrophic scar [43]. By summarizing publications, Liu et al. provided several targets in this pathway that were available for pharmaceutical development [44].

Instead of using miRNA mimics/agomir, Tgfbr1 was directly inhibited by siRNA-loaded liposomes in the pre-clinical section of our study. One major concern prohibiting mimics/agomir from clinical application is the lack of comprehensive knowledge of downstream targets [45]. Besides, mimics/agomir is synthesized chemically in lab, which may increase the risk of the off-target effect [46]. Therefore, recent years witnesses the efforts in making modified/bioengineered miRNA agents to better recapitulate the properties of natural RNAs in live cells [47]. On ground of this, we finally gave up the idea of directly loading miRNA agomir.

On the contrary, siRNA is already approved by FDA with several ongoing clinical trials. Since Tgfbr1 is proved to be targeted by miR-142, si-Tgfbr1 was tested in the translational section of the paper eventually. siRNAs have high efficiency and specificity, but the large size and hydrophilicity prevent them from entering cell independently [48]. Moreover, the negative charge makes siRNAs unstable in circulation [49]. Since cationic liposomes can be easily prepared with high translational potential and good biocompatibility as well as high encapsulation efficiency [50, 51], this vector was chosen by us to overcome the shortcomings of siRNAs in vivo.

The development of adhesive capsulitis is currently poorly understood. When combined with RCT, researches are fewer. Tsai et al. noticed elevated level of type 1 cannabinoid (CB1) receptor in those with joint stiffness, while treating tenocytes with CB1 agonist or antagonist could induce or reduce the expression of interleukin-1β [52]. Wang et al. found that, the level of miR-29a in both the serum and subacromial bursa was reduced in RCT patients with stiffness than those without, and this miRNA could mitigate inflammation and fibrosis in human tenocytes, improving tendon fibrosis in mouse model [53]. Likewise, we also noticed a differential expression of miRNAs in tissue and circulation.

On the other hand, we failed to pinpoint a differential expression of miR-29a in our cohort. The phenomenon could be explained by two reasons. First, human bursa specimen and tenocytes were harvested by Wang et al. [53], while we collected human joint capsule sample and CDFs. Second, we only measured the level of circulating exosomal miRNAs, while Wang et al. examined the whole level of miR-29a/b/c in serum [53]. This divergence indicates that multiple mechanisms may be involved to induce adhesive capsulitis.

Since its first report in 1934, adhesive capsulitis has been bothering patients, physicians, and scientists for almost a century with limited treatments of high efficiency. Various mechanisms, including metabolic, inflammatory, and even immunological signaling, are reported [2], indicating the complexity of this condition. In this research, we tried to investigate this disorder via analyzing circulating exosomal miRNAs. By understanding the natural reaction of organism, potential targets may be unveiled. At first, we expected to find some new molecules. Unintendedly, we finally found a simple way to antagonize adhesive capsulitis, which was based on a classical signaling pathway that has been investigated for countless times. This result reminds us to get back to nature.

The reliability of our findings was strengthened by various factors. First, to understand the pathology, primary CDFs from patients with or without adhesive capsulitis were harvested. These primary cells maintain the pro-fibrotic features even after passaged [29], and therefore could provide referential value for clinic. As the features of an activated phenotype, cell viability, proliferation, as well as contraction and migration ability are enhanced [42, 54]. The up-regulation of various markers including Col 1, α-SMA, and VIM, were also examined. In addition, to serve as the basis of experiments in mice, the effect of exosomes or miRNAs was also tested on NIH3T3 murine fibroblasts. As a pre-clinical model, mice shoulders could mimic the clinical features of shoulder stiffness, such as joint capsule thickening, cell infiltration, and loss of ROM [35]. Importantly, the mice were first subject to immobilization surgery, and then delivered with interventions one week later. This design ensured a fibrotic status of the index shoulder, making our data more applicable to clinic status.

On the other hand, the data should also be interpreted with caution. Firstly, although we confirmed the beneficial effect of miR-142 against fibrosis, bioinformatic analysis indicated that this miRNA may have multiple functions. More experiments are needed to elucidate the involvement of other signaling pathways. Secondly, to guarantee the concentration, we directly injected agents into the shoulder joint of mice. The impact of the agents on rotator cuff tendon should be discussed in the future. If appropriate, targeted delivery method against CDFs can be developed. Thirdly, activity level, diet, lifestyles, and many other factors can have an influence on the level of circulating miRNAs [55]. What is the effect of these confounders on RCT or adhesive capsulitis should be investigated in the future. Additionally, we only used CDFs within the first three passages for experiments in vitro, which means that only limited amount of CDFs can be harvested from one patient. Therefore CDFs from multiple individuals were harvested for experiments. This patient-to-patient variation may introduce bias and cause a fluctuation of data. Finally, we noticed an effusion of agents around the location of joint capsule, which was indicated by in vivo imaging. This phenomenon suggested that there could be a leakage of agents from the joint capsule, which might also induce bias to the data. Further researches are needed to establish an accurate injection portal.

## Conclusion

In conclusion, our research found that, in RCT population, circulating exosomes from patients with adhesive capsulitis could inhibit fibrosis. This effect was related to the miR-142 highly enriched in these exosomes. By targeting Tgfbr1, the pro-fibrotic pathway was abolished by miR-142. Enlightened by this mechanism, si-Tgfbr1 was loaded into liposomes, and we proved the anti-fibrotic potential of this agent.

## Supplementary Information

**Additional file 1:** **Fig. S1.** Flowchart of animal experiments. One week after model establishment, two intra-articular injections were delivered weekly. Samples were harvested at 21d from model establishment. **Fig. S2.** Fibrotic changes was observed in human capsule samples in group S. Representative immunofluorescence images of α-SMA (A) and Col 1 (B) in the human capsular samples. Bar=100 μm. **Fig. S3.** Exo-NS and Exo-S were uptaken by CDFs. Typical image of the uptake of PKH67-labeled Exo-NS and Exo-S (green) by CDFs (Dapi blue) and negative control (Dye-only). Bar=25 μm. **Fig. S4.** TGF-β highly expressed in human capsule samples in group S than group NS. Typical immunofluorescent images illustrated the expression of TGF-β (green) in the human shoulder capsular samples. Scale bar=100 μm. **Fig. S5.** Exo-NS had no significant influence on TGF-β mediated promotion of cell viability. Viability of CDFs by CCK-8 analysis. ***: P < 0.001 compared to group NC. **Fig. S6.** Exo-S were uptaken by NIH3T3 cells. Typical picture of the uptake of PKH67-labeled Exo-S (green) by NIH3T3 (Dapi blue) and negative control (Dye-only). Bar=25 μm. **Fig. S7.** Exo-S relieved fibrogenesis of NIH3T3 cells induced by TGF-β. A and B: Protein level of Col 1 and α-SMA in NIH3T3 cells under different stimulations and quantification; C and E: Proliferation of NIH3T3 cells and quantification. Bar=180 μm; D and F: Migration ability of cells (red dotted line indicated the border of wound) and quantification; G: Viability of NIH3T3 cells probed by CCK-8 assay. ***: P < 0.001 compared to group NC; ****: P < 0.0001 compared to group NC; #: P<0.05 compared to group TGF-β; ##: P<0.01 compared to group TGF-β; ###: P<0.001 compared to group TGF-β; ####: P<0.0001 compared to group TGF-β. **Fig. S8.** miR-142 inhibited CDFs fibrogenesis induced by TGF-β. A and C: Proliferation of CDFs and quantification. Bar=180 μm; B and D: Migration ability of CDFs (orange dotted line indicated the border of the wound) and quantification; E: Viability of CDFs determined by CCK-8 assay; F and G: Protein level of p-Smad2/3 and t-Smad2/3 in CDFs and quantification; H and I: Collagen contraction ability of CDFs and quantification (red dotted circle indicated the collagen). **: P < 0.01 compared to group NC; ***: P < 0.001 compared to group NC, ****: P < 0.0001 compared to group NC; ##: P < 0.01 compared to group TGF-β + NC mimics; $: P < 0.05 compared to group NC inhibitor; $$: P < 0.01 compared to group NC inhibitor. **Fig. S9.** Anti-fibrotic effect of Exo-S depended on miR-142 in vitro. A and B: Proliferation of CDFs and quantification. Bar=180 μm; C: Viability of CDFs was determined using CCK-8 assay; D and E: Immunofluorescent staining of α-SMA in CDFs (red forα-SMA and blue for nucleus) and quantification. Bar=25μm. *: P < 0.05 compared to group TGF-β, **: P < 0.01 compared to group TGF-β; #: P < 0.05 compared to group TGF-β + Exo-S. **Fig. S10.** Expression of miR-142 and Tgfbr1 in patients shoulder capsules. The expression of miR-142 (A) and Tgfbr1 (B) in patients’ capsule samples were lower in group S than in group NS. **: P < 0.01; ****: P < 0.0001. **Fig. S11.** The efficacy of siRNAs for knocking down the expression of Tgfbr1 in NIH3T3 cells. A and B: The knock-down efficacy of three siRNAs and quantification; C and D: Proliferation of NIH3T3 cells and quantification. Bar=180 μm; E: Relative expression of fibrotic markers in NIH3T3 cells; F and G: Migration ability of NIH3T3 cells (red dotted line indicated the border of wound) and quantification. *: P < 0.05 compared to group NC; **: P < 0.01 compared to group NC; ***: P < 0.001 compared to group NC; ****: P < 0.0001; #: P < 0.05 compared to group TGF-β; ##: P < 0.01 compared to group TGF-β; ###: P < 0.001 compared to group TGF-β. **Fig. S12.** Tracing of DiO-labeled liposomes in vitro. Liposomes entered NIH3T3 cells when co-culturing for 30min and 60min. **Fig. S13.** Tracing of liposomes in vivo. A: 24h following intra-articular injection, DiO-labeled liposomes were viewed in the cells of capsule tissue. Bar=25μm; B: Immediately, one day, and two days after DiR-labeled liposomes injection, fluorescent signaling was viewed at the injection site (white circle). **Fig. S14.** Toxicity evaluation in vivo. Typical HE picture of the major organs (liver, lung, intestine, heart, spleen, and kidney) in mice model with or without si-Tgfbr1-loading liposomes injections. **Table S1.** Primary antibodies used in the experiments. **Table S2.** The sequence of miRNAs and siRNAs. **Table S3.** Primers used in the study. **Table S4.** Basic characteristics of patients enrolled. 

## Data Availability

The exosomal microRNA sequencing data was uploaded in the GEO dataset (GSE182896).

## Abbreviations

S: Stiffness
NS: Non-stiffness
3ʹ-UTR: 3ʹ-Untranslated Region
CDFs: Capsular derived fibroblasts
CCK-8: Cell counting kit-8
CDS: Coding sequence
FACS: Flow cytometry analysis
KEGG: Kyoto Encyclopedia of Genes and Genomes
GO: Gene Ontology
miRNAs: MicroRNAs
NC: Negative control
qPCR: Quantitative polymerase chain reaction
RCT: Rotator cuff tear
SEM: Scanning electron microscope
TEM: Transmission electron microscope
