# Supplementary material for: si-Tgfbr1-loading liposomes inhibit shoulder capsule fibrosis via mimicking the protective function of exosomes from patients with adhesive capsulitis
Source: Biomater Res. 2022 Aug 19;26:39. doi: 10.1186/s40824-022-00286-2 (PMC9389696; doi:10.1186/s40824-022-00286-2)
Supplement: Supplementary file 1 — Additional file 1: Fig. S1. Flowchart of animal experiments. One week after model establishment, two intra-articular injections were delivered weekly. Samples were harvested at 21d from model establishment. Fig. S2. Fibrotic changes was observed in human capsule samples in group S. Representative immunofluorescence images of α-SMA (A) and Col 1 (B) in the human capsular samples. Bar=100 μm. Fig. S3. Exo-NS and Exo-S were uptaken by CDFs. Typical image of the uptake of PKH67-labeled Exo-NS and Exo-S (green) by CDFs (Dapi blue) and negative control (Dye-only). Bar=25 μm. Fig. S4. TGF-β highly expressed in human capsule samples in group S than group NS. Typical immunofluorescent images illustrated the expression of TGF-β (green) in the human shoulder capsular samples. Scale bar=100 μm. Fig. S5. Exo-NS had no significant influence on TGF-β mediated promotion of cell viability. Viability of CDFs by CCK-8 analysis. ***: P < 0.001 compared to group NC. Fig. S6. Exo-S were uptaken by NIH3T3 cells. Typical picture of the uptake of PKH67-labeled Exo-S (green) by NIH3T3 (Dapi blue) and negative control (Dye-only). Bar=25 μm. Fig. S7. Exo-S relieved fibrogenesis of NIH3T3 cells induced by TGF-β. A and B: Protein level of Col 1 and α-SMA in NIH3T3 cells under different stimulations and quantification; C and E: Proliferation of NIH3T3 cells and quantification. Bar=180 μm; D and F: Migration ability of cells (red dotted line indicated the border of wound) and quantification; G: Viability of NIH3T3 cells probed by CCK-8 assay. ***: P < 0.001 compared to group NC; ****: P < 0.0001 compared to group NC; #: P<0.05 compared to group TGF-β; ##: P<0.01 compared to group TGF-β; ###: P<0.001 compared to group TGF-β; ####: P<0.0001 compared to group TGF-β. Fig. S8. miR-142 inhibited CDFs fibrogenesis induced by TGF-β. A and C: Proliferation of CDFs and quantification. Bar=180 μm; B and D: Migration ability of CDFs (orange dotted line indicated the border of the wound) and quantification; E: [file 40824_2022_286_MOESM1_ESM.docx]

**si-Tgfbr1-loading liposomes inhibit shoulder capsule fibrosis via mimicking the protective function of exosomes from patients with adhesive capsulitis**

Fig. S1: Flowchart of animal experiments.

One week after model establishment, two intra-articular injections were delivered weekly. Samples were harvested at 21d from model establishment.

**
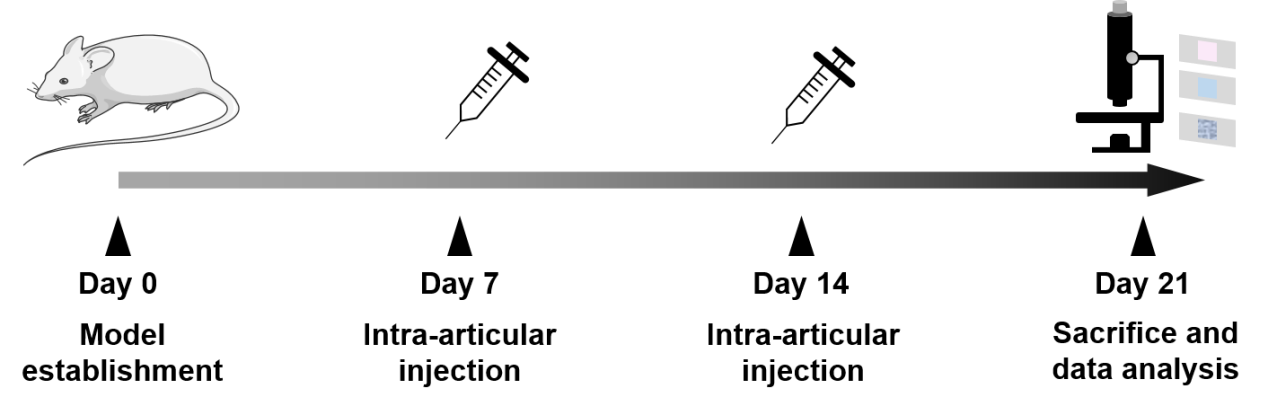
**

Fig. S2: Fibrotic changes was observed in human capsule samples in group S.

Representative immunofluorescence images of α-SMA (A) and Col 1 (B) in the human capsular samples. Bar=100 μm.


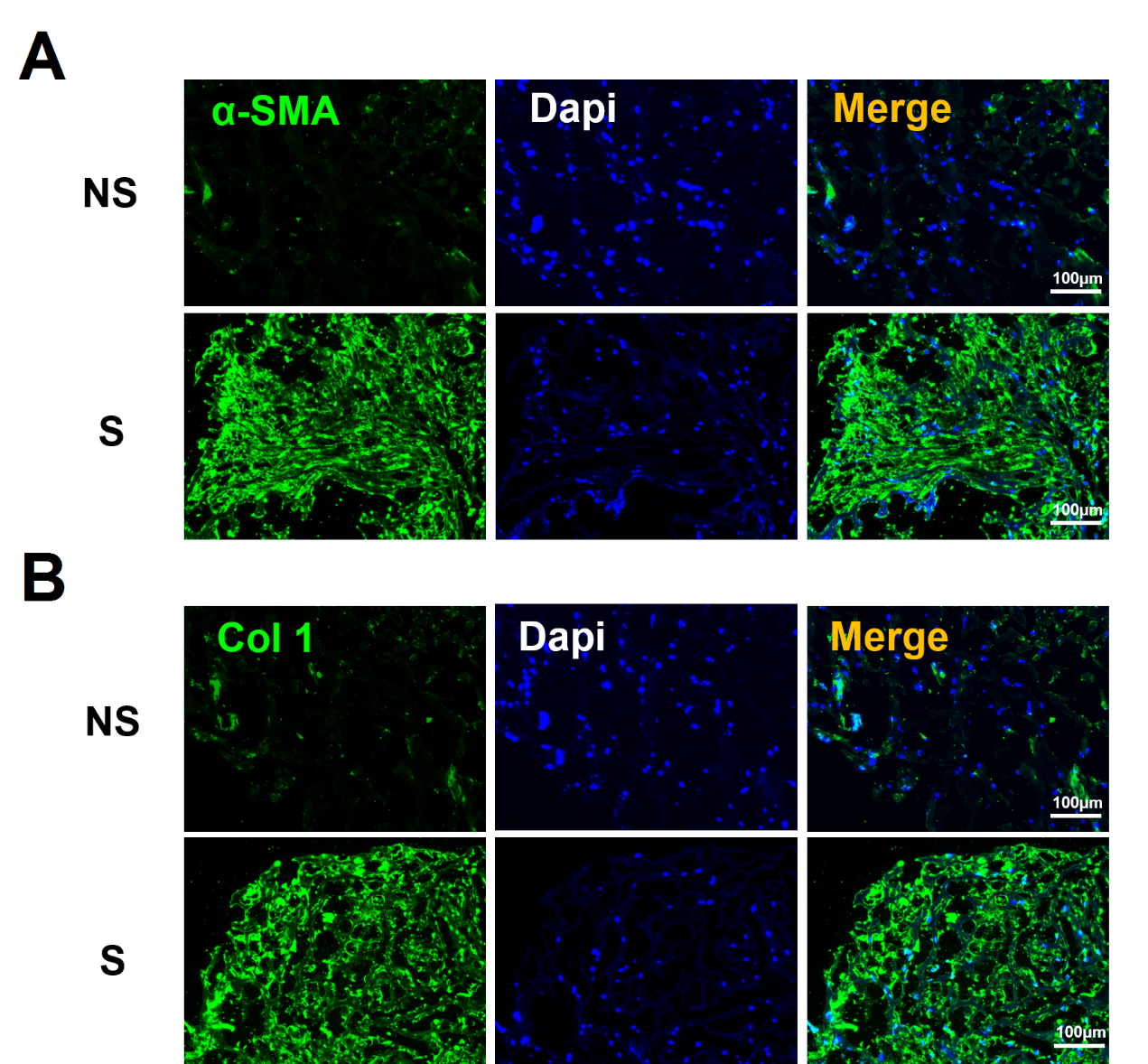


Fig. S3: Exo-NS and Exo-S were uptaken by CDFs.

Typical image of the uptake of PKH67-labeled Exo-NS and Exo-S (green) by CDFs (Dapi blue) and negative control (Dye-only). Bar=25 μm.


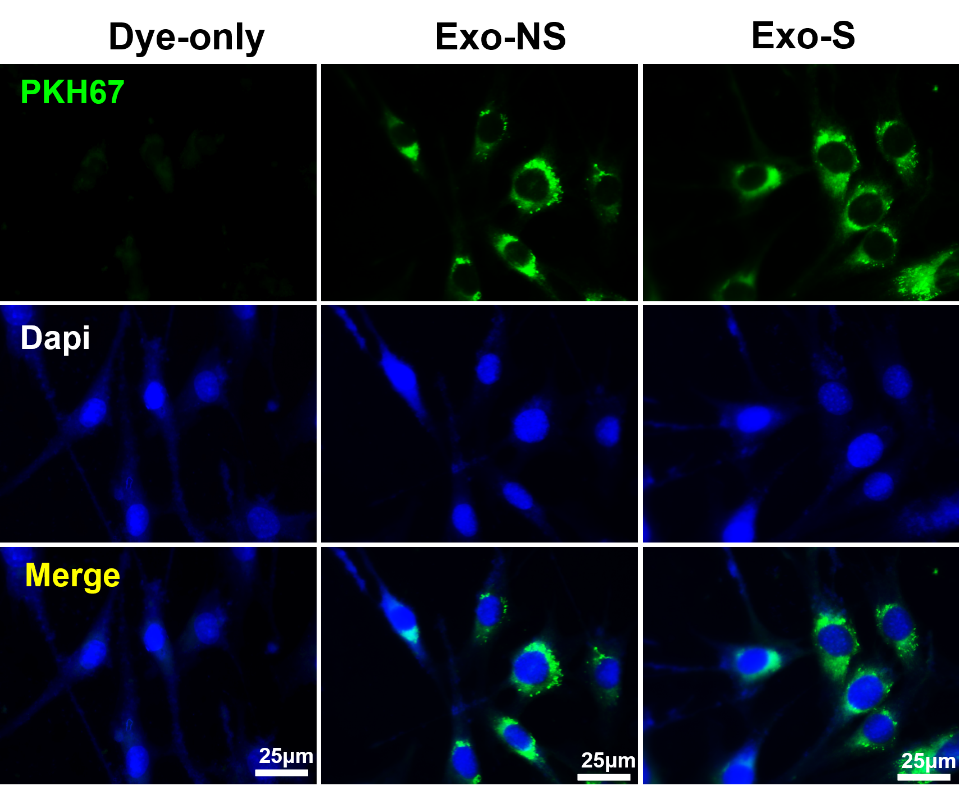


Fig. S4: TGF-β highly expressed in human capsule samples in group S than group NS.

Typical immunofluorescent images illustrated the expression of TGF-β (green) in the human shoulder capsular samples. Scale bar=100 μm.


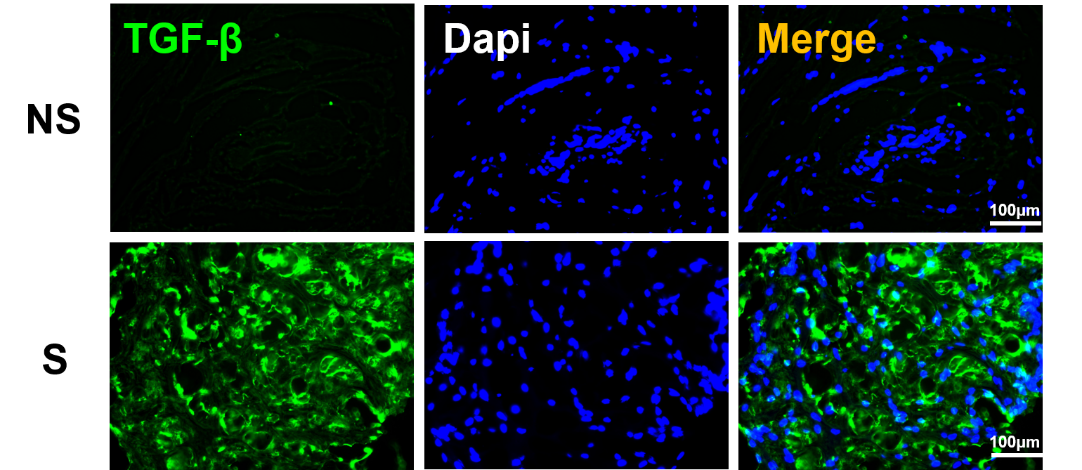


Fig. S5: Exo-NS had no significant influence on TGF-β mediated promotion of cell viability.

Viability of CDFs by CCK-8 analysis. ***: P < 0.001 compared to group NC.


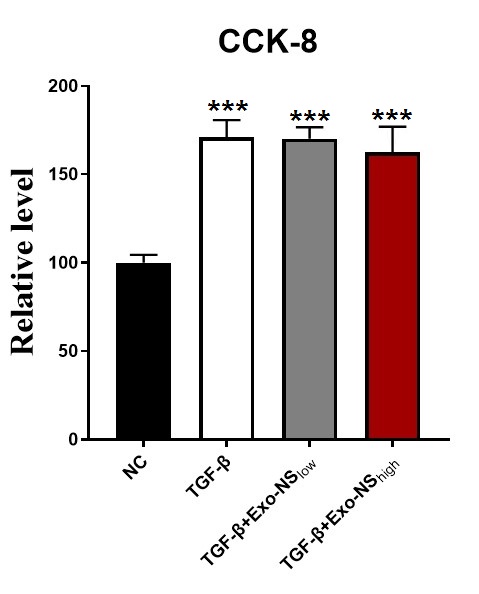


Fig. S6: Exo-S were uptaken by NIH3T3 cells.

Typical picture of the uptake of PKH67-labeled Exo-S (green) by NIH3T3 (Dapi blue) and negative control (Dye-only). Bar=25 μm.


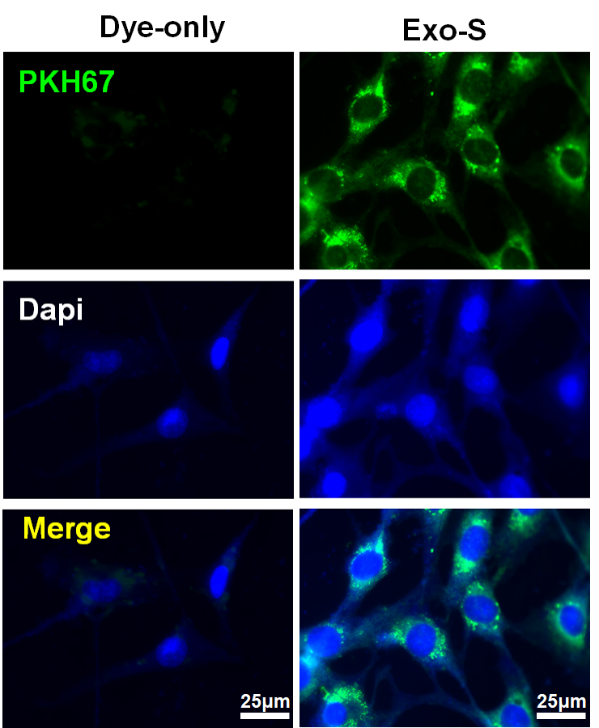


Fig. S7: Exo-S relieved fibrogenesis of NIH3T3 cells induced by TGF-β.

A and B: Protein level of Col 1 and α-SMA in NIH3T3 cells under different stimulations and quantification; C and E: Proliferation of NIH3T3 cells and quantification. Bar=180 μm; D and F: Migration ability of cells (red dotted line indicated the border of wound) and quantification; G: Viability of NIH3T3 cells probed by CCK-8 assay. ***: P < 0.001 compared to group NC; ****: P < 0.0001 compared to group NC; #: P<0.05 compared to group TGF-β; ##: P<0.01 compared to group TGF-β; ###: P<0.001 compared to group TGF-β; ####: P<0.0001 compared to group TGF-β.


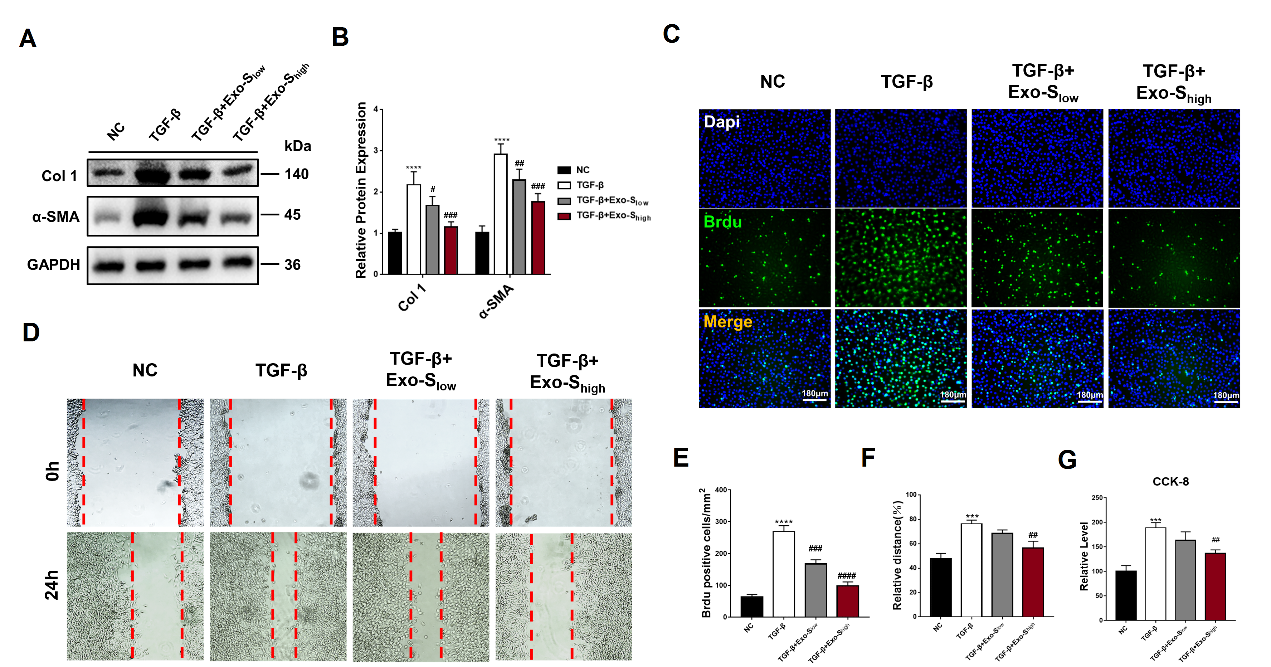


Fig. S8: miR-142 inhibited CDFs fibrogenesis induced by TGF-β.

A and C: Proliferation of CDFs and quantification. Bar=180 μm; B and D: Migration ability of CDFs (orange dotted line indicated the border of the wound) and quantification; E: Viability of CDFs determined by CCK-8 assay; F and G: Protein level of p-Smad2/3 and t-Smad2/3 in CDFs and quantification; H and I: Collagen contraction ability of CDFs and quantification (red dotted circle indicated the collagen). **: P < 0.01 compared to group NC; ***: P < 0.001 compared to group NC, ****: P < 0.0001 compared to group NC; ##: P < 0.01 compared to group TGF-β + NC mimics; $: P < 0.05 compared to group NC inhibitor; $$: P < 0.01 compared to group NC inhibitor.


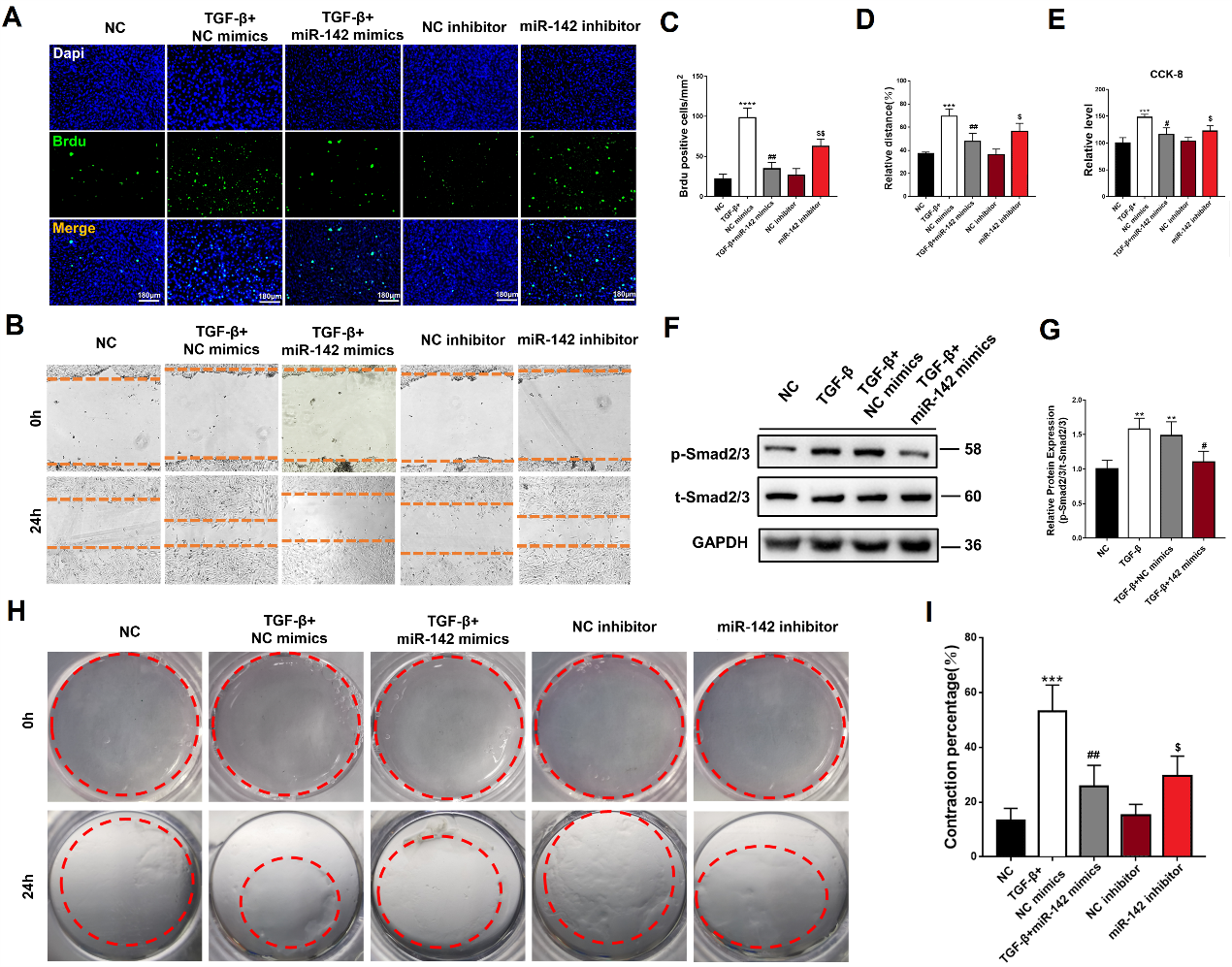


Fig. S9: Anti-fibrotic effect of Exo-S depended on miR-142 in vitro.

A and B: Proliferation of CDFs and quantification. Bar=180 μm; C: Viability of CDFs was determined using CCK-8 assay; D and E: Immunofluorescent staining of α-SMA in CDFs (red forα-SMA and blue for nucleus) and quantification. Bar=25μm. *: P < 0.05 compared to group TGF-β, **: P < 0.01 compared to group TGF-β; #: P < 0.05 compared to group TGF-β + Exo-S.


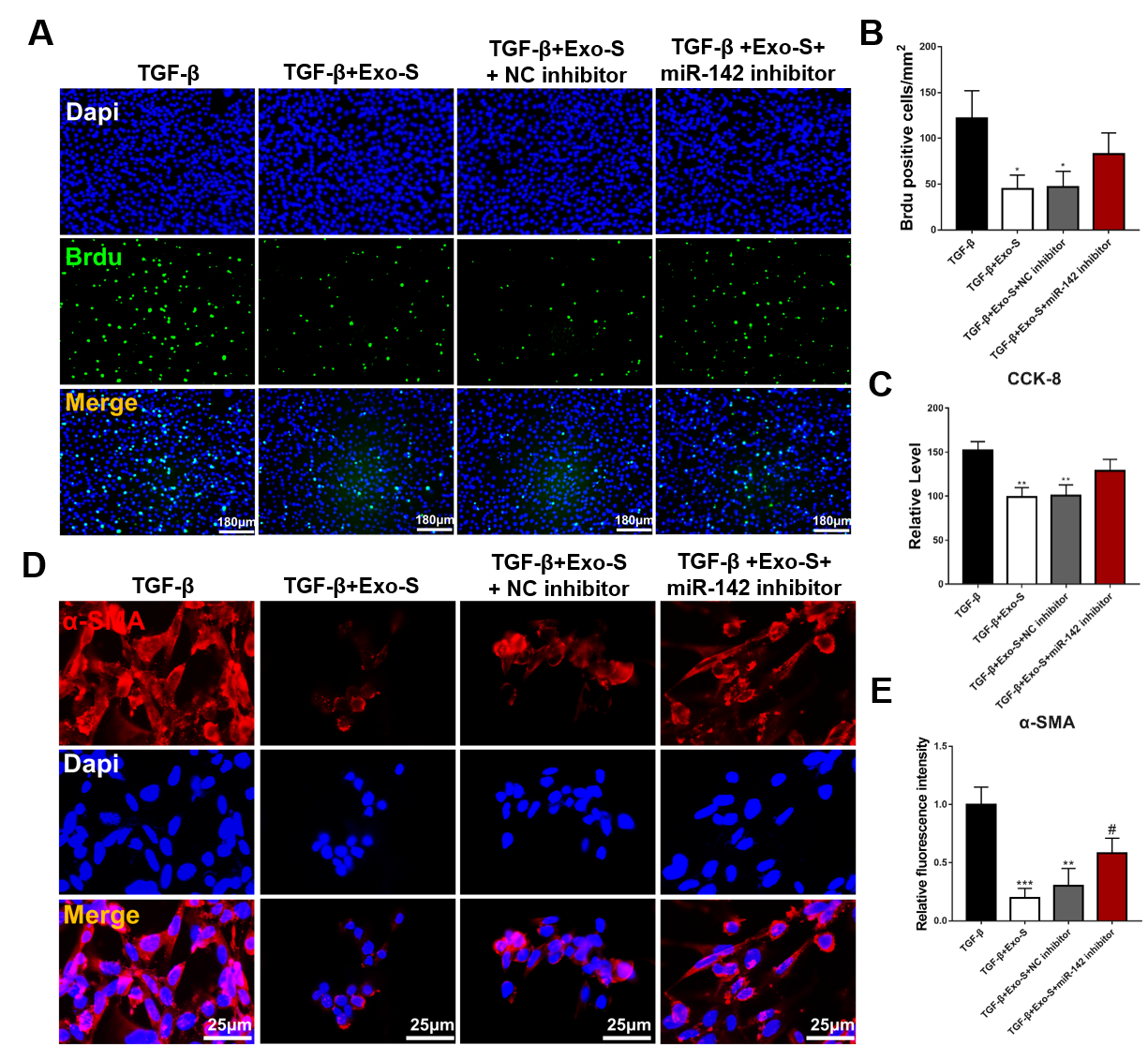


Fig. S10: Expression of miR-142 and Tgfbr1 in patients shoulder capsules.

The expression of miR-142 (A) and Tgfbr1 (B) in patients’ capsule samples were lower in group S than in group NS. **: P < 0.01; ****: P < 0.0001.


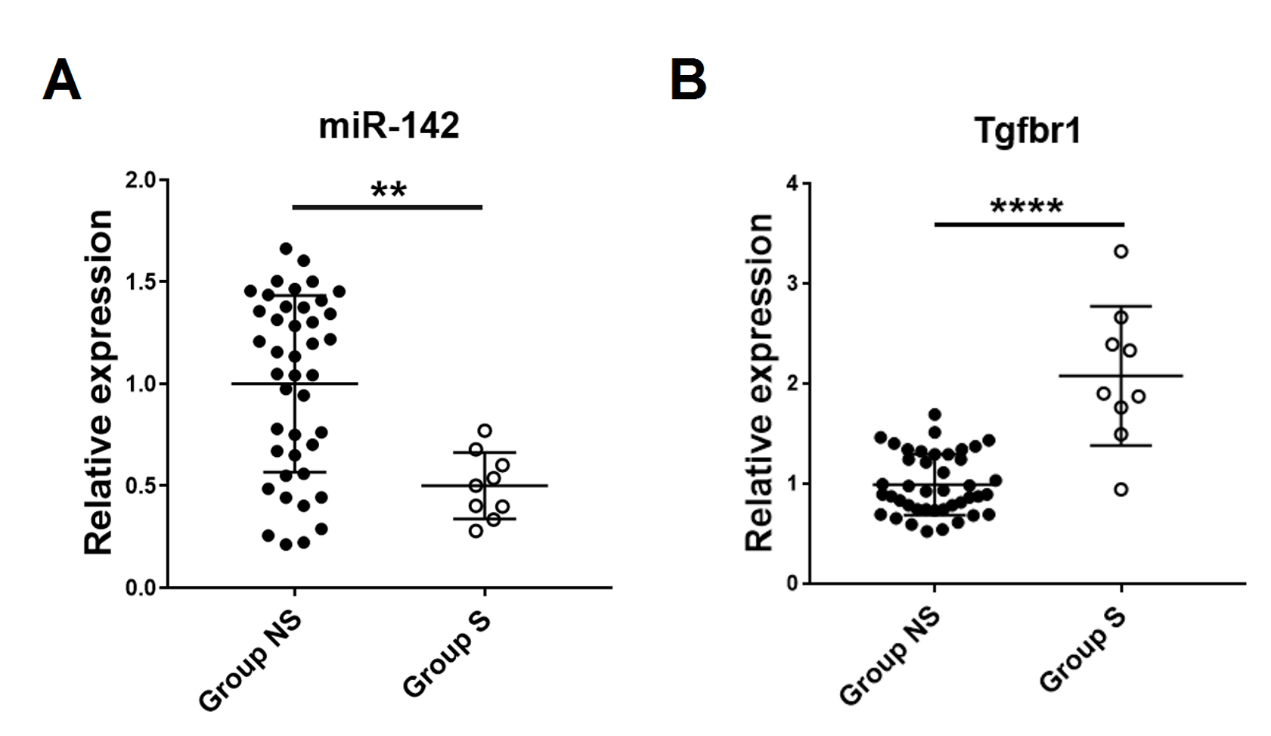


Fig. S11: The efficacy of siRNAs for knocking down the expression of Tgfbr1 in NIH3T3 cells.

A and B: The knock-down efficacy of three siRNAs and quantification; C and D: Proliferation of NIH3T3 cells and quantification. Bar=180 μm; E: Relative expression of fibrotic markers in NIH3T3 cells; F and G: Migration ability of NIH3T3 cells (red dotted line indicated the border of wound) and quantification. *: P < 0.05 compared to group NC; **: P < 0.01 compared to group NC; ***: P < 0.001 compared to group NC; ****: P < 0.0001; #: P < 0.05 compared to group TGF-β; ##: P < 0.01 compared to group TGF-β; ###: P < 0.001 compared to group TGF-β.


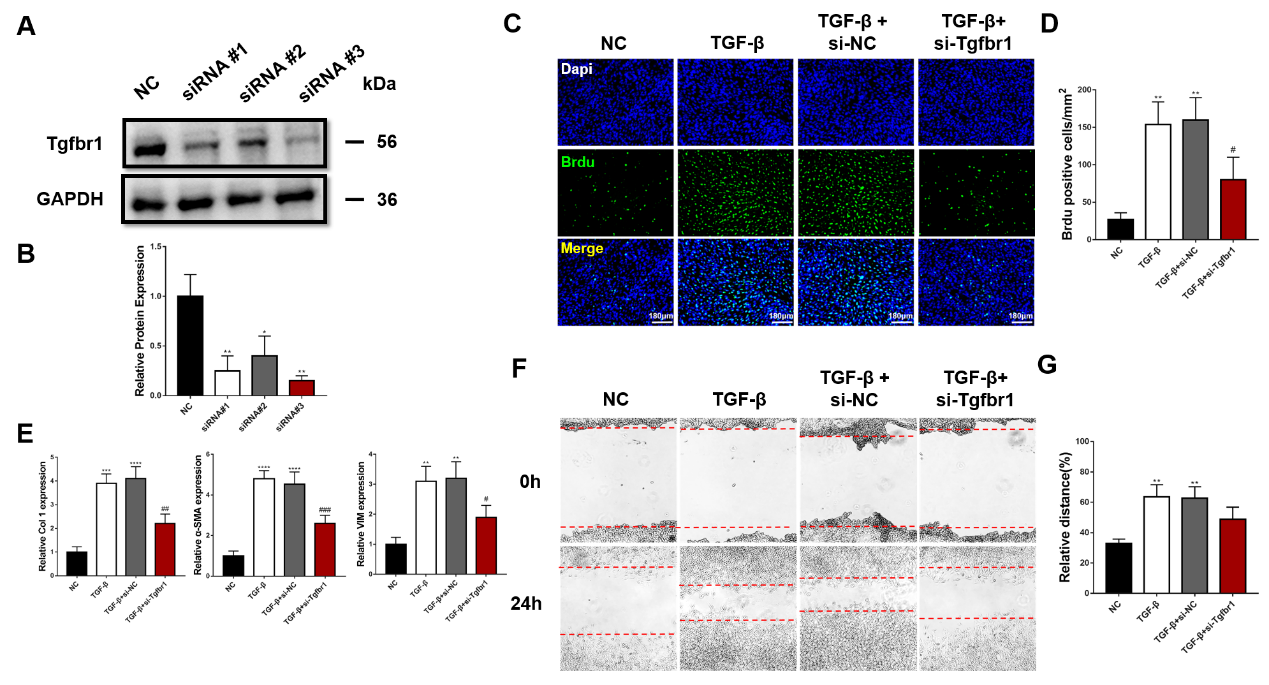


Fig. S12: Tracing of DiO-labeled liposomes in vitro.

Liposomes entered NIH3T3 cells when co-culturing for 30min and 60min.


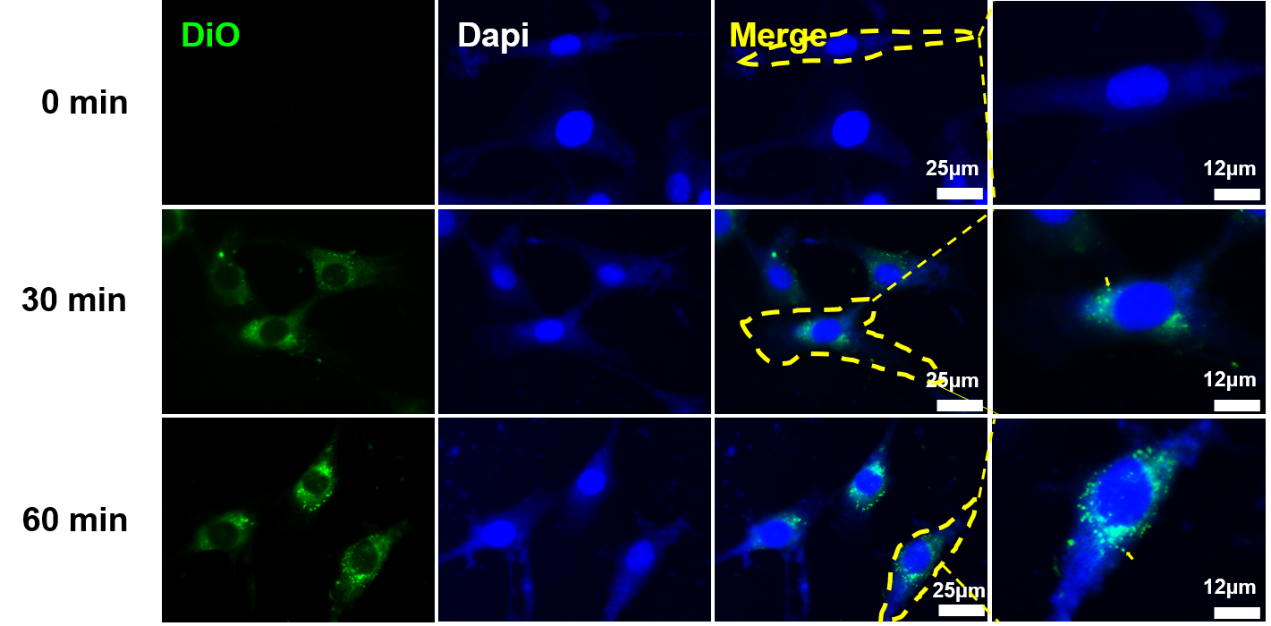


Fig. S13: Tracing of liposomes in vivo.

A: 24h following intra-articular injection, DiO-labeled liposomes were viewed in the cells of capsule tissue. Bar=25μm; B: Immediately, one day, and two days after DiR-labeled liposomes injection, fluorescent signaling was viewed at the injection site (white circle).


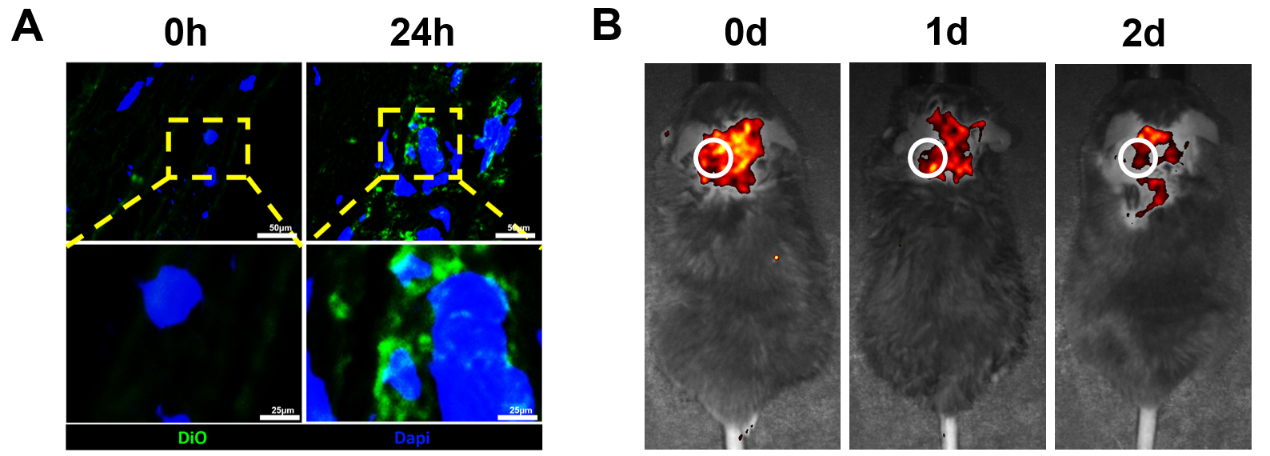


Fig. S14: Toxicity evaluation in vivo.

Typical HE picture of the major organs (liver, lung, intestine, heart, spleen, and kidney) in mice model with or without si-Tgfbr1-loading liposomes injections.


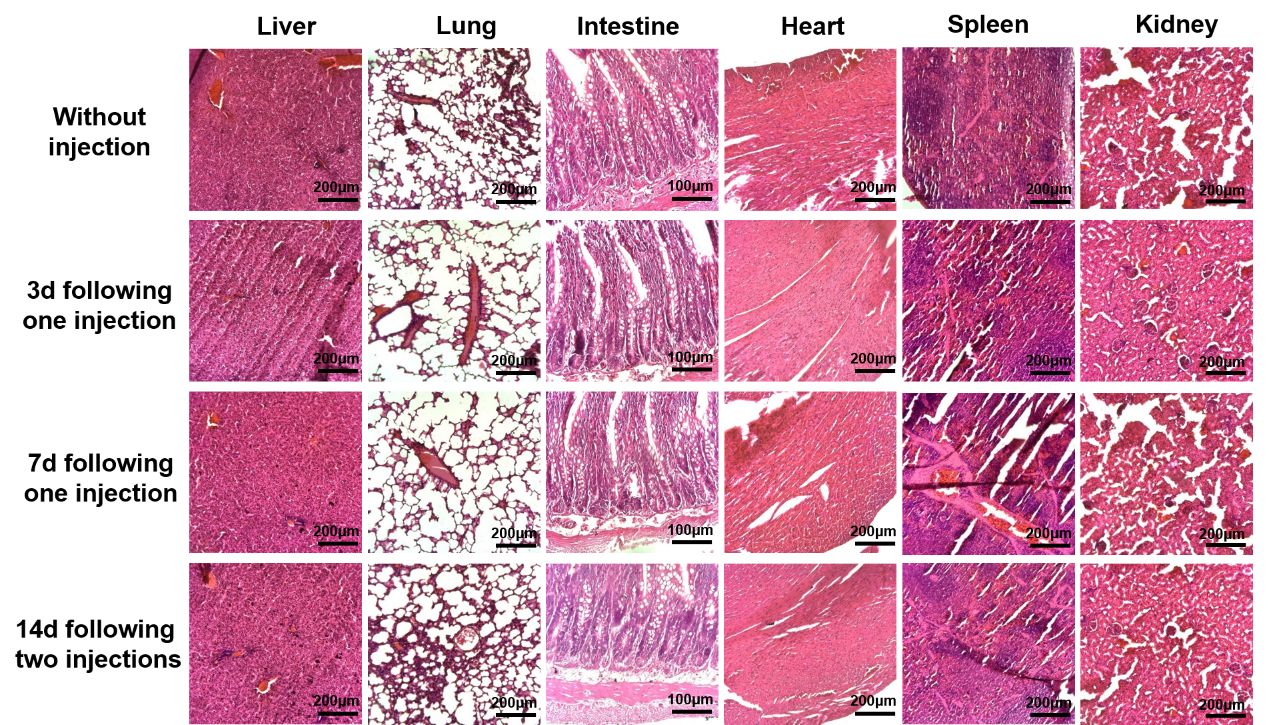


**Table S1. Primary antibodies used in the experiments**

| Antibody | Source | Catalog No. | Type | kD |
| --- | --- | --- | --- | --- |
| CD63  CD9  Alix  HSP60  Albumin | Affinity  Affinity  Affinity  Affinity  Abcam | AF5117  AF5139  ab275377  AF0184  ab207327 | Rabbit mAb  Rabbit mAb  Rabbit mAb  Rabbit mAb  Rabbit mAb | 47  23  95  60  69 |
| TGF-β1 | Abcam | ab215715 | Rabbit mAb | 44 |
| t-Smad2/3 | Affinity | AF6367 | Rabbit mAb | 60 |
| p-Smad2/3  α-SMA | Affinity  Affinity | AF3367  AF1032 | Rabbit mAb  Rabbit mAb | 58  42 |
| α-SMA | Abcam | ab7817 | Mouse mAb | 42 |
| Collagen 1  Vimentin  Tgfbr1  GAPDH | Affinity  Affinity  Abcam  Affinity | AF7001  AF7013  ab235178  AF7021 | Rabbit mAb  Rabbit mAb  Rabbit mAb  Rabbit mAb | 140  53  56  37 |

**Table S2. The sequence of miRNAs and siRNAs**

| Name | Sense sequence(5'-3') | Antisense sequence(5'-3') |
| --- | --- | --- |
| si-Tgfbr1 (human) | GGGUCUGUGACUACAACAU dTdT | AUGUUGUAGUCACAGACCC dTdT |
| si-Tgfbr1 (mouse) (siRNA-1) | CCAGGACCAUUGUGUUACA dTdT | UGUAACACAAUGGUCCUGG dTdT |
| si-Tgfbr1 (mouse) (siRNA-2) | GCAAGGCUGUAGCAUUGAU dTdT | AUCAAUGCUACAGCCUUGC dTdT |
| si-Tgfbr1 (mouse) (siRNA-3) | GGUUCACUUUGAAUGUACU dTdT | AGUACAUUCAAAGUGAACC dTdT |
| NC siRNA | UUCUCCGAACGUGUCACGU | ACGUGACACGUUCGGAGAA |
| miR-142 mimics | UGUAGUGUUUCCUACUUUAUGGA | UCCAUAAAGUAGGAAACACUACA |
| miR-142 inhibitor | UCCAUAAAGUAGGAAACACUACA |  |
| NC mimics | UUUGUACUACACAAAAGUACUG | CAGUACUUUUGUGUAGUACAAA |
| NC inhibitor | CAGUACUUUUGUGUAGUACAAA |  |

**Table S3. Primers used in the study**

| Primer Name | Primers |
| --- | --- |
| Tgfbr1 | F: TCAGCTCTGGTTGGTGTCAG  R: ATGTGAAGATGGGCAAGACC |
| Col 1 | F: GCTCCTCTTAGGGGCCACT  R: CCACGTCTCACCATTGGGG |
| α-SMA | F: GTCCCAGACATCAGGGAGTAA  R: TCGGATACTTCAGCGTCAGGA |
| VIM | F: CGGCTGCGAGAGAAATTGC  R: CCACTTTCCGTTCAAGGTCAAG |
| miR-7-1-3p | F: TTATTTTTTTTTAGGTGGTGGT  R: ACACAACCTTATAATCCCAATT |
| miR-4488 | F: AGGGGGCGGGCTCC  R: TGGTGTCGTGGAGTCG |
| miR-122-5p | F: GGGCTGGAGAGAGACAAT  R: GTGCAGGGTCCGAGGT |
| miR-142-3p | F: GTCGTATCCAGTGCAGGG  R: GTGCAGGGTCCGAGGT |
| U6 | F: CTCGCTTCGGCAGCACA  R: AACGCTTCACGAATTTGCGT |
| GAPDH | F: CCGTTGAATTTGCCGTGA  R: TGATGACCCTTTTGGCTCCC |
| Cel-miR-39 | F: GTGCAGGGTCCGAGGT  R: GTGCAGGGTCCGAGGT |

**Table S4. Basic characteristics of patients enrolled**

| Items | group NS | group S | P value |
| --- | --- | --- | --- |
| Number (male) | 42 (15) | 9 (2) | 0.436 |
| Age (year) | 55.4±10.3 | 60.8±6.9 | 0.141 |
| Duration (month) | 18.10±37.04 | 6.44±3.47 | 0.354 |
| Trauma | 25 | 4 | 0.407 |
| Right side | 29 | 5 | 0.436 |
| Height (cm)  Weight (Kg) | 162.6±6.5  61.3±9.6 | 161.2±8.7  54.9±7.4 | 0.5836  0.0663 |
| Flexion (degree) | 153.41±17.09 | 93.75±6.96 | <0.001 |
| External rotation at side (degree) | 57.44±15.97 | 16.25±6.50 | <0.001 |
| Internal rotation at back* | 10.37±2.03 | 15.88±1.17 | <0.001 |

*: Vertebra level was numbered serially 1 to 12 for the 1st to 12th thoracic vertebrae, 13 to 17 for the 1st to 5th lumbar vertebrae, and 18 for below the sacral region. NS: non-stiffness. S: stiffness.
